# Targeting Mouse Double Minute 2: Current Concepts in DNA Damage Repair and Therapeutic Approaches in Cancer

**DOI:** 10.3389/fphar.2020.00631

**Published:** 2020-05-07

**Authors:** Wen Li, Xinhao Peng, Jinyi Lang, Chuan Xu

**Affiliations:** ^1^ Cancer Clinical Research Center & Integrative Cancer Center, Sichuan Cancer Hospital & Institute Sichuan Cancer Center, School of Medicine, University of Electronic Science and Technology of China, Chengdu, China; ^2^ Radiation Oncology Key Laboratory of Sichuan Province, Sichuan Cancer Hospital & Institute Sichuan Cancer Center, School of Medicine, University of Electronic Science and Technology of China, Chengdu, China

**Keywords:** MDM2, P53, DNA damage repair, genomic instability, clinical inhibitor

## Abstract

Defects in DNA damage repair may cause genome instability and cancer development. The tumor suppressor gene p53 regulates cell cycle arrest to allow time for DNA repair. The oncoprotein mouse double minute 2 (MDM2) promotes cell survival, proliferation, invasion, and therapeutic resistance in many types of cancer. The major role of MDM2 is to inhibit p53 activity and promote its degradation. In this review, we describe the influence of MDM2 on genomic instability, the role of MDM2 on releasing p53 and binding DNA repair proteins to inhibit repair, and the regulation network of MDM2 including its transcriptional modifications, protein stability, and localization following DNA damage in genome integrity maintenance and in MDM2-p53 axis control. We also discuss p53-dependent and p53 independent oncogenic function of MDM2 and the outcomes of clinical trials that have been used with clinical inhibitors targeting p53-MDM2 to treat certain cancers.

## Introduction

**Figure 1:**
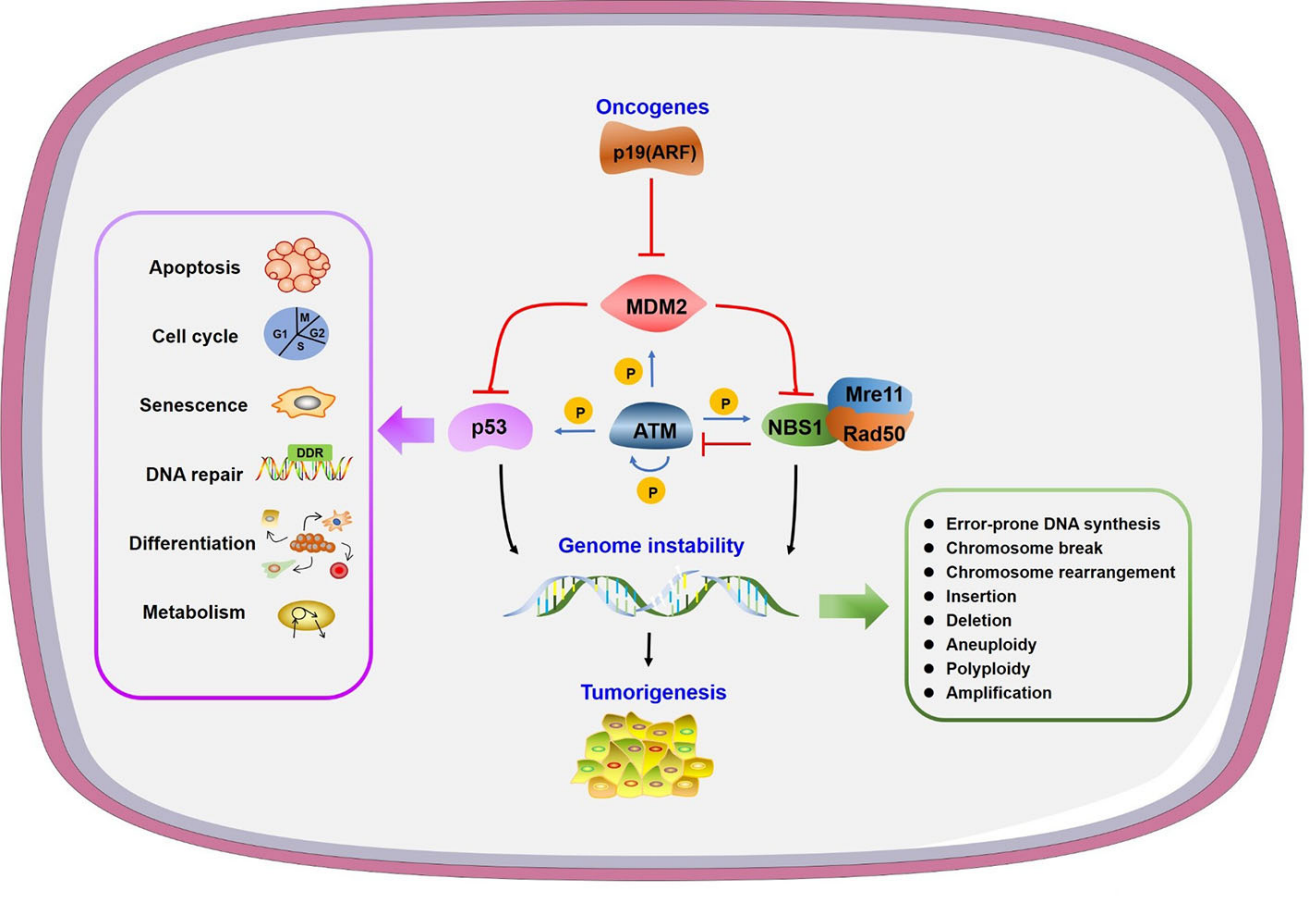
Genome instability and other consequences caused by mouse double minute 2 (MDM2). Oncogene p19 (ARF) interacts with MDM2 to prevent its function. MDM2 can inhibit both p53 and NBS1 to regulate many physiological processes or/and lead to genome instability, in which ATM plays a central phosphorylation regulatory role.

Genomic instability is a hallmark of cancer and is regulated by a balance between DNA damage and repair (Aguilera and Garcia-Muse, 2013). The consequences of genomic instability include error-prone DNA synthesis, chromosome copy number variations and chromosomal structural aberrations (Jasin, 2000; Ganem et al., 2007) (**Figure 1**). Conversely, unrepaired damage may cause transcription and replication arrest, inducing cell death and senescence (Hoeijmakers, 2009).

In order to address the threat posed by genome changes, cells have evolved a kinase cascade signal transduction pathway called DNA damage response (DDR) (Harper and Elledge, 2007). The initial step in active DDR is a rapid accumulation of DNA repair proteins to damage sites, which serve as a scaffold to recruit downstream factors (Soutoglou and Misteli, 2010). Mis-regulated DDR may increase accumulation of unrepaired DNA lesions and trigger aberrant cell proliferation, leading to malignance diseases (Jackson and Bartek, 2009).

Tumor suppressor p53 accumulates at DNA damage sites directly in both sequence-specific and non-sequence-specific manner to play a protective role in DDR (Lee et al., 1995; Liu and Kulesz-Martin, 2001) (**Figure 1**). P53 deficiency leads to reduced repair activity and decreased cell survival after UV-induced DNA damage, indicating the participation of p53 in nucleotide excision repair (NER) (Smith et al., 1995). P53 also takes part in both global genome NER and transcription coupled NER through DNA polymerase δ and ϵ, respectively (Mirzayans et al., 1996). Interaction between p53 with apyrimidinic endonuclease APE1/Ref-1 and the regulatory function of OGG1, MUTYH, and 3-methyladenine DNA glycosylase by p53 all indicate the function of p53 in base excision repair (BER). Among the two major pathways in double-strand break repair (DSBR), p53 interacts with both non-homologous end-joining (NHEJ) proteins and the promoter of homologous recombination (HR) protein RAD51 to regulate its expression (Williams and Schumacher, 2016).

The E3 ligase MDM2 (murine double minute 2) contains 491 amino acids and is a major negative regulator of p53 (**Figure 1**). MDM2 controls activity, subcellular localization, and stability of p53 (Eischen and Lozano, 2014). Increased MDM2 expression is commonly observed in different types of cancers, indicating its oncogenic function (Eischen and Lozano, 2014). MDM2 interacts with large and diverse substrates through complex structures, post-translational modifications, and diverse isoforms to function as a significant hub in different signal pathways (Fahraeus and Olivares-Illana, 2014). The major role of p53 as a tumor suppressor relies on its transcriptional activity to regulate target genes in diverse biological pathways (Sullivan et al., 2018). MDM2 binds p53 directly through N-terminal transactivation domain to inhibit p53 transactivation (Momand et al., 1992; Oliner et al., 1993). MDM2 also forms homodimers to ubiquitinate p53 leading to p53 proteasomal degradation (Haupt et al., 1997; Kubbutat et al., 1997). In this review, we highlight the specific regulation network of MDM2 in DDR and discuss the potential clinical applications of MDM2 antagonists and the perspective of developing novel combination strategies.

## Genome Instability Induced by MDM2

As the negative regulator of p53, MDM2’s function in cell survival versus cell death is complex and depends on the extent of DNA damage and the capacity of repair. The abnormal MDM2-p53 regulatory loop and its corresponding delayed DDR provide an additional layer for genomic instability control. The interaction of MDM2 with p53 and Mre11-Rad50-Nbs1 complex indicates MDM2 affects genomic instability in both p53-dependent and p53-independent manner.

### MDM2 Affects Genomic Instability in p53-Dependent Manner

Given that MDM2 binds with p53 to inhibit its transactivation and that it induces p53 protein degradation, it is reasonable to assume that MDM2 regulates genome instability through p53 directly. Oncogene Myc activates p19 (ARF)-MDM2 pathway to coordinate p53-dependent checkpoint and apoptotic program (Zindy et al., 1998; Eischen et al., 1999). ARF interacts with MDM2 to guide its degradation and prevents negative feedback of p53/MDM2, thus preventing p53 degradation (Zhang et al., 1998; Weber et al., 1999). A high level of MDM2 was identified in advanced stage breast ductal carcinomas and squamous cell carcinomas with wild-type p53. Overexpression of MDM2, and/or p53 deletion or mutation induced centrosome hyper-amplification and chromosome instability in these cancer cells (Carroll et al., 1999). A study of lymphoma transformation showed that MDM2 overexpression in B cells led to a reduced susceptibility to p53-dependent apoptosis through inhibition of p53 and p21 (Wang et al., 2008). MDM2 transgenic mice also showed increased chromosome breaks, chromosome fusions, aneuploidy, and polyploidy (Lushnikova et al., 2011). It has also been shown that p53 disruption by MDM2 overexpression activates the intra S-phase checkpoint to inhibit DNA replication origin firing, causing replication fork instability (Frum et al., 2014; Primo and Teixeira, 2019).

### MDM2 Affects Genomic Instability in p53-Independent Manner

MDM2 also affects genomic instability independent of p53. It was found that mammary epithelial cells underwent multiple rounds of S phase without cell division to form increased polyploidy in response to MDM2 overexpression regardless of p53 expression level in mice (Lundgren et al., 1997). Mre11/Rad50/NBS1 (MRN) complex is a central sensor in DSBR. Mre11 and NBS1 mutant mice exhibit checkpoint defects and chromosomal instability (Stracker et al., 2004). MDM2 associates with MRN complex through the direct interaction with NBS1 independent of p53, thus leads to recruitment of MDM2 at DNA damage sites with delayed DNA repair and compromised DNA integrity (Alt et al., 2005). Interaction of MDM2 with NBS1 inhibits DNA repair and leads to increased chromosome breaks and transformation efficiency in p53 deficient cells (Bouska et al., 2008). With small molecular inhibitors targeting the interaction of p53 with MDM2, a p53 independent function of MDM2 in NBS1 regulation is found and worthy of considering in cancer drug design (Bouska and Eischen, 2009). In addition to binds NBS1, it has been reported that MDM2 ubiquitinates transcription factor HBP1 to facilitate its degradation, thus preventing the transcriptional inhibition role of HBP1 to its target genes to induce genomic instability like global DNA hypermethylation and histone hypermethylation (Cao et al., 2019).

## Inhibitory Role of MDM2 in DNA Repair

MDM2 is regulated downstream of DNA damage by several mechanisms such as inactivation by post-translational modification and destruction p53-MDM2 interaction to relieve p53 inhibition. Increased p53 level leads to cell senescence, cell cycle arrest, and apoptosis. In the meanwhile, the interaction of MDM2 with DNA repair complex MRN exists and is independent of p53 state (Eischen, 2017). The accumulated unrepaired DNA breaks further activate p53 and facilitate alterations such as chromosome translocations, gene fusions, increased micronuclei, and gene amplification, which are common causes of cancer malignancy (Morgan et al., 1998; Bunting and Nussenzweig, 2013). MDM2 is in turn transcriptionally regulated by p53 to form a feed-back loop to maintain cellular homeostasis under genetic stress.

### Release of p53 After DNA Damage

**Figure 2:**
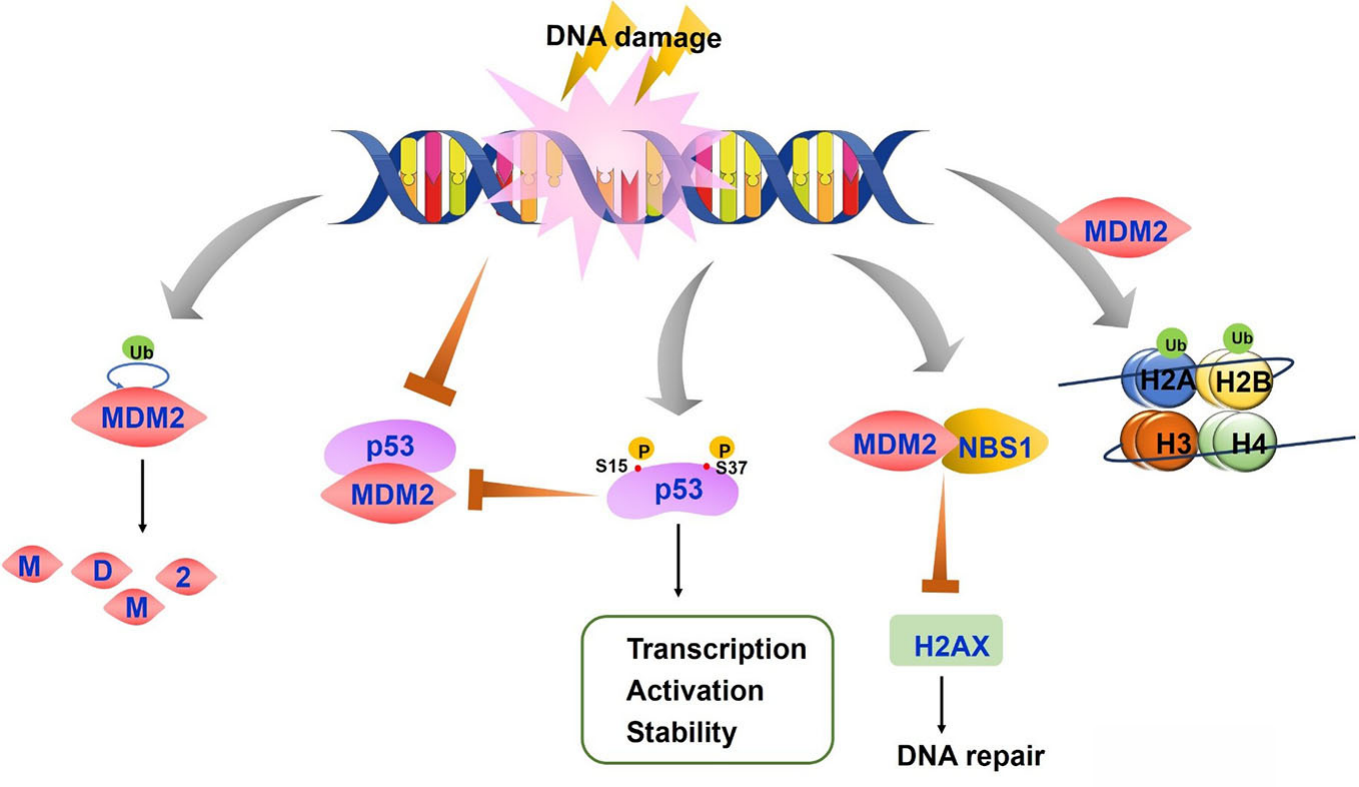
The regulation of p53-mouse double minute 2 (MDM2) axis after DNA damage. On the condition of DNA damage, both MDM2 auto-degradation and the disruption of p53-MDM2 interaction lead to stable and active p53. P53 is also phosphorylated at Ser15 and Ser37, resulting in the reduced p53-MDM2 interaction and increased transcription, activation and stability of p53 protein. MDM2-NBS1 association delays H2AX phosphorylation and inhibits DNA repair. MDM2 induces histones H2A and H2B monoubiquitylation, which affects chromatin structure and accessibility.

The regulatory function of MDM2 on p53 after DNA damage is complex as it depends on the type of damage, p53 substrates, and various modifications on p53 and MDM2 proteins. The key step of p53 activation after DNA damage and other genetic stress results in the disruption of p53-MDM2 interaction and p53 release (**Figure 2**). The inhibitory role of MDM2 on p53 includes blocking p53 transcriptional activation and regulating p53 protein level. Disruption of p53-MDM2 interaction alone is enough for p53 stabilization and activation even without DNA damage (Bottger et al., 1997). The MDM2 binding sites in p53 amino terminus overlap with the binding sites in p53 that interact with the transcription machinery. MDM2 competes with transcription factors such as TFIID and TAFII31 for p53 binding to block p53 mediated transcription (Lin et al., 1994; Lu and Levine, 1995; Thut et al., 1995). A study also indicated that MDM2 directly repressed basal transcriptional machinery through its inhibitory domain (Thut et al., 1997). NEDD8 has a similar modification with ubiquitination, which can conjugate with p53 in a MDM2 dependent manner to repress p53 transcription (Xirodimas et al., 2004). MDM2 also leads to a proteasomal degradation of p53. Moreover, expression of MDM2 significantly inhibits p53 accumulation after DNA damage. These results indicate a post-translational regulation under stress conditions (Haupt et al., 1997; Kubbutat et al., 1997).

Treatment with DNA damaging agents induce p53 phosphorylation at serine 15 and 37 which causes conformation changes in p53, resulting in reduced interaction of p53 with MDM2 (Shieh et al., 1997). P53 acetylation was also reported to block p53-MDM2 interaction and which enable p53-dependent cell growth arrest and apoptosis under stress conditions (Tang et al., 2008). In addition, cells exhibit increased p53 phosphorylation by ATM, ATR, and other kinases after DNA damage with UV light and other agents, leading to p53 stabilization and enhanced DNA binding. The higher levels of p53 further activate transcription of downstream targets including MDM2 (Kruse and Gu, 2009). As the repair process progresses, increased MDM2 decreases p53 expression and activity through the above mechanisms, forming a regulatory feedback loop to keep p53 and MDM2 protein at a basal level (Maltzman and Czyzyk, 1984; Freedman et al., 1999). The consequences of p53 regulation by MDM2 also depend on MDM2 activity. Low levels of MDM2 activity lead to p53 mono-ubiquitination and nuclear export whereas high levels promote p53 polyubiquitination and nuclear degradation (Li et al., 2003).

### Interaction of MDM2 With MRN Complex

Mre11/Rad50/Nbs1 (MRN) complex is essential in the maintenance of genome, meiosis, and telomere (D’Amours and Jackson, 2002). Mre11 exhibits 3′–5′ double strand DNA exonuclease and endonuclease activity and joints two broken ends using their microhomology domains (Paull and Gellert, 1999). Rad50 is a structural maintenance of chromosome (SMC) related protein, which functions in bridging DSB ends together to facilitate Mre11-dependent DSB end processing (Williams et al., 2007). NBS1 recognizes DSB ends and transduces cell cycle checkpoint signals through ATM-induced epigenetic modification. NBS1 also functions in chromosome remodeling in response to DSB (Williams et al., 2007). After ATM activation and HR repair initiation by MRN, nucleases such as CtIP, Exo1, and Dna2/BLM, cause DSB end resection to form 3′-overhangs, which are coated by RPA and Rad51 to facilitate strand exchange (Mladenov et al., 2016).

Although MDM2 promotes genomic instability and cancer development through the inhibition of p53, studies have also identified MDM2 function independent of p53 (Bouska and Eischen, 2009). MDM2 directly interacts with NBS1 through a 31-amino-acid region and is recruited to damage sites independent of p53 and ARF, a negative regulator of MDM2 (Alt et al., 2005) (**Figure 2**). MDM2-NBS1 association delays H2AX and ATM-S/TQ phosphorylation and inhibits DNA repair (Bouska et al., 2008). Additionally, MDM2 can bind other proteins involved in DNA replication or repair, such as DNA polymerase ϵ (Asahara et al., 2003).

### Other Regulatory Mechanisms of MDM2 Following DNA Damage

Mice with a hypomorphic allele in MDM2 gene revealed increased p53 transcriptional activation and apoptosis (Mendrysa et al., 2003). Increased MDM2 protein level as a result of a single nucleotide polymorphism led to a decreased p53 signal transduction and higher tumor risk (Bond et al., 2004). All these evidences indicate that the amount of MDM2 protein dictates the physiological consequences of p53 activation. Further studies found that DNA damage induced MDM2 auto-degradation, which conversely stabilized and activated p53 (Stommel and Wahl, 2004). The half-life of MDM2 decreased after treatment with DNA damage agents neocarzinostatin (NCS), UV irradiation, and BCNU, while p53 stability and activation increased. This process is reversible and the half-life of MDM2 increases which corrects with unstable and inactive p53 when stress was eliminated (Stommel and Wahl, 2005).

Other studies demonstrated that MDM2 modulate DDR process through its association with and modification of chromatin, especially the promoter region of p53 target genes. Histone modification has long been known to affect transcription and DNA repair by influencing chromatin structure and accessibility, which is important for the recruitment of chromatin remodeling factors. MDM2 had been reported to interact with histones directly and induce monoubiquitylation of histones H2A and H2B *in vitro* and of H2B *in vivo* (Minsky and Oren, 2004). In addition to that, the p53-MDM2 interaction may change p53 conformation and inhibit its binding to DNA. This function of MDM2 is mediated by its central acidic domain which binds to histone methyl transferase Suv39h1. The Suv39h1-MDM2 interaction restores p53 conformation allowing DNA binding of p53-MDM2-Suv39h1 complex (Cross et al., 2011). On the contrary, MDM2 was also reported to polyubiquitinate Suv39h1 at lysine 87 and to promote its degradation (Bosch-Presegue et al., 2011). This could be attributed to differences in cell context and experimental conditions (Wienken et al., 2017). A p53-independent function of MDM2 in gene repression under stress conditions through chromatin modification warrants further investigation.

## MDM2 Regulation in Response to DNA Damage

MDM2 binds N terminal of p53 to inhibit its transcription and promote its proteasomal degradation. MDM2 is also regulated by p53 to form an autoregulatory loop. Since MDM2 gene amplification and protein overexpression are found widely in human cancers, investigating the MDM2 related regulatory network under DNA damage is essential to understand its biological function as an oncogene and to identify novel targets for cancer therapy.

### Regulation of MDM2 Expression

MDM2 gene can be transcribed from two independent promoters, P1 and P2. The P1 promoter transcribes from the first exon but without exon 2. P1 promoter carries out basal transcription and its activation does not need p53. P2 promoter is located within the first intron which includes two p53-binding sites and the transcriptional activation of P2 depends on p53 (Barak et al., 1994; Zauberman et al., 1995). Since the identification of increased expression of MDM2 variant in a range of human cancers and decreased expression in normal tissue in 1996, more than 72 kinds of MDM2 splice variants have been observed in both cancer and normal cells (Sigalas et al., 1996; Rosso et al., 2014). Some of these variants are specifically spliced in response to DNA damage (Jeyaraj et al., 2009). However, their molecular mechanisms remain unknown.

The most common splice variants of MDM2 are MDM2-A (ALT2), MDM2-B (ALT1), and MDM2-C (ALT3). Compared to the full length MDM2 (MDM2-FL), which consists of 12 exons, MDM2-A lacks exon 4–9, MDM2-B lacks exon 4–11, and MDM2-C lacks exon 5–9. All these three variants lack p53 binding site at N terminal while they retain the C terminal RING domain, which facilitates their interaction with MDM2-FL (Huun et al., 2017). Based on such structural features, MDM2-A has been characterized to be a p53 activator. MDM2-A expression exhibits enhanced p53 activity and decreased transformation in p53-null setting (Volk et al., 2009). Activated p53/p21 pathway and increased cyclins D1 and E were discovered after MDM2-A expression (Sanchez-Aguilera et al., 2006). MDM2-B is frequently expressed in various cancer types including ovarian cancer, bladder cancer, astrocytic cancer, breast cancer, and giant cell tumors of bone (Sigalas et al., 1996; Matsumoto et al., 1998; Evdokiou et al., 2001; Lukas et al., 2001). MDM2-B binds and sequesters full-length MDM2 in the cytoplasm and promotes p53 transcription by inhibiting interaction of MDM2-FL with p53 (Evans et al., 2001). Using a specific human MDM2-C antibody, high expression of endogenous MDM2-C was detected in cancer cell lines and in cancer tissues. Unlike MDM2-A and MDM2-B, MDM2-C had no effect on p53 degradation and transcription regulation but showed p53-independent transformation property (Okoro et al., 2013).

Studies have identified a single nucleotide polymorphism (T/G SNP309) in MDM2 promoter region. This variant exhibit increased affinity toward the transcriptional activator Sp1, resulting in higher levels of MDM2 RNA and protein (Bond et al., 2004). In MDM2 SNP309 cells, p53 binds chromatin but cannot be activated (Arva et al., 2005). Overexpressed MDM2 with SNP309 is associated with increased risk of renal cancer development and worse patient prognosis in esophageal squamous cell carcinoma and B-cell chronic lymphocytic leukemia (Hong et al., 2005; Hirata et al., 2007; Gryshchenko et al., 2008). MDM2 expression can be regulated by miRNAs induced by p53. Wild type p53 was identified in many multiple myeloma cases which induced the expression of miR-192, 215, and 194 leading to the downregulation of MDM2 (Pichiorri et al., 2010).

### Regulation of MDM2 Modification

**Figure 3:**
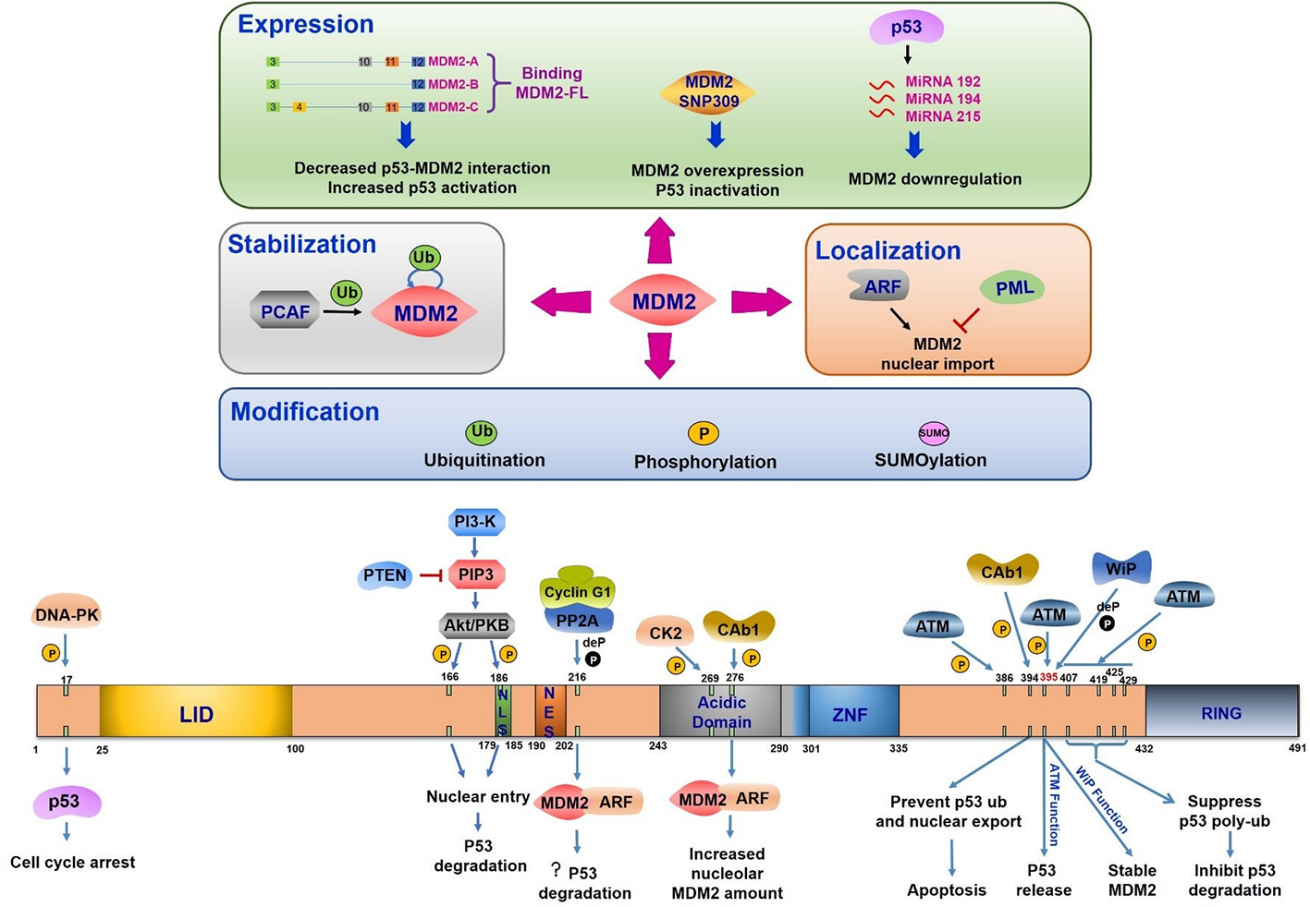
Mouse double minute 2 (MDM2) regulation under DNA damage. The upper half part reflects the regulation of MDM2 expression, stabilization, modification, and location. All the three splice variants can interact with MDM2-FL, leading to the disruption of p53-MDM2 interaction and increased p53 activation. The single nucleotide polymorphism (T/G SNP309) exhibits increased MDM2 expression and p53 inhibition. P53 induces miR-192, 215, and 194 expression, which leads to MDM2 downregulation. The PCAF induced ubiquitination or auto-ubiquitination in MDM2 relate to its stability. ARF-induced MDM2 nucleus importation increases MDM2 mediated p53 response. PML plays the opposite role. MDM2 modification includes its ubiquitination, phosphorylation, and SUMOylation. The bottom half part indicates domains of MDM2, its phosphorylation and dephosphorylation sites, the related kinases or phosphatases, and the corresponding consequences. LID, Lid domain; NLS, nuclear localization site; NES, nuclear export site; Acidic Domain, central acidic domain; ZNF, zinc finger domain; RING, RING domain; ? (question mark), this function is still needed to be confirmed.

The structural domains of MDM2 include (1) an N terminal lid domain (25–100 aa), a hydrophobic pocket controlling p53 binding, (2) a nuclear localization site (179–185 aa, NLS), (3) a nuclear export site (190–202 aa, NES), (4) a central acidic domain (243–301 aa), (5) a zinc finger domain (290–335 aa), and (6) a RING domain (432–491 aa), which is in charge of E3 ligase activity (Fahraeus and Olivares-Illana, 2014) (**Figure 3**). MDM2 can be modified multisite phosphorylation/dephosphorylation, ubiquitination and SUMOylating (**Figure 3**).

#### Phosphorylation

MDM2 is frequently phosphorylated especially in the central acidic domain (Blattner et al., 2002). Many kinases are reported to regulate MDM2 phosphorylation which controls its downstream activation or inhibition. The multiple phosphorylation sites within MDM2 can be divided into three groups (1) serine residues at 157, 166, 186, and 188 all of which are present near nuclear localization sites and nuclear export sites, (2) serine residues at 240, 242, 246, 253, 256, 260, 262, and 269 which is the phosphorylated cluster in the absence of any stress, and (3) serine 17, threonine 216, tyrosine 276, tyrosine 394, and serine at 395 and 407, all of which are phosphorylated under DNA damage stimulation (Meek and Hupp, 2010).

The most important mediators in DDR are phosphatidylinositol 3-kinase-like protein kinase (PIKKs) family like ATM (ataxia telangiectasia mutated), ATR (ATM and Rad3-related), and DNA-PK (DNA-dependent protein kinase). DNA double strand breaks activate ATM and DNA-PK to induce downstream transduction. ATR is activated by the recruitment of RPA-coated single strand breaks with a partner protein ATRIP (Ciccia and Elledge, 2010). Based on the presence of serine and threonine residues, which make up about 20% of all amino acids in MDM2 protein, MDM2 is a suitable phosphorylation substrate (Meek and Knippschild, 2003). The posttranslational modification of N-terminal region in both MDM2 and p53 can disrupt their interaction under cellular stress (Appella and Anderson, 2001). The 16–24 residues of MDM2 were reported to form a lid crossing over p53 binding sites. This region functions in stabilizing MDM2 protein but weakly inhibits p53 binding (McCoy et al., 2003). Interestingly, phosphorylated states in this region showed a significant influence in disrupting MDM2-p53 interaction. Following DNA damage, DNA-PK phosphorylates MDM2 at serine 17 and inhibits its interaction with p53, leading to p53 activation and G1 cell cycle arrest (Mayo et al., 1997). ATM mediated substrate phosphorylation is an early response to DNA damage. MDM2 is phosphorylated by ATM at serine 395 (serine 394 in mouse) facilitating p53 release and accumulation in response to ionizing radiation and radiomimetic agents but not to UV radiation (de Toledo et al., 2000; Maya et al., 2001). Other residues (Ser386, Ser395, Ser407, Thr419, Ser425, and Ser429) located in C terminals of MDM2 have been identified to be phosphorylated by ATM after DNA damage in a redundant manner (Cheng et al., 2009). The phosphorylation of these sites inhibits oligomerization of RING domain resulting in suppression of p53 poly-ubiquitination and degradation (Cheng et al., 2011). Activation of a nuclear tyrosine kinase c-Abl is another response to ionizing radiation. This kinase is phosphorylated by ATM and is involved in cell cycle control (Baskaran et al., 1997; Brown and McCarthy, 1997; Shafman et al., 1997). Following DNA damage, c-Abl binds MDM2 directly, phosphorylates MDM2 at tyrosine 394, and prevents p53 ubiquitination and its nuclear export. This c-Abl induced MDM2 tyrosine 394 phosphorylation triggered increased p53 transcription and decreased degradation leading to p53-mediated apoptosis (Sionov et al., 2001; Goldberg et al., 2002). Additionally, tyrosine 276 was found to be phosphorylated by c-Abl in response to DNA damage *in vitro*. This modification enhanced MDM2 interaction with ARF with an increased expression of nucleolar MDM2 (Dias et al., 2006). These striking observations pointed out that c-Abl phosphorylation site is adjacent to ATM phosphorylation site indicating that ATM regulates MDM2 phosphorylation both in direct and indirect manner.

Recent studies indicate that many growth factors and cytokines are involved in cancer progression by regulating cell proliferation and apoptosis. The serine/threonine protein kinase Akt/PKB is an essential signal transducer downstream of phosphatidylinositol 3-kinase (PI3-kinase) (Lawlor and Alessi, 2001). MDM2 can be phosphorylated at serine 166 and serine 186 by Akt/PKB which results nuclear translocation from cytoplasm (Mayo and Donner, 2001). In addition to that, HER-2/neu-mediated Akt activation phosphorylates MDM2 to enhance p300 interaction and inhibits p19ARF interaction, resulting in increased degradation of p53 and blocked cytotoxic effect of DNA damage agents in cancer cells (Zhou et al., 2001). Following stimulation with growth factors, PI3-kinase is activated and generates second messenger phosphatidylinositol (3,4,5)-trisphosphate PIP3 (Vanhaesebroeck and Alessi, 2000). A lipid phosphatase and tumor suppressor protein PTEN dephosphorylates PIP3 to disrupt this signal transduction. The mechanism is illustrated by the fact that high level of PTEN or PI3-K inhibitor dephosphorylates serine 166 and serine 186 of MDM2 and blocks its nuclear entry leading to an increased cellular p53 level and activity. Meanwhile, p53 further promotes PTEN expression to form a positive feedback loop in response to cellular stress. The PI3-K-Akt-MDM2 axis connects two important tumor suppressor PTEN and p53 to regulate cell survival, drug resistance and tumorigenesis (Mayo and Donner, 2001; Mayo et al., 2002; Mayo and Donner, 2002; Ogawara et al., 2002). The protein kinase CK2 (CK2) is required for cell viability and cell cycle progression. CK2 is another protein kinase that phosphorylates MDM2 at serine 269 (Gotz et al., 1999; Hjerrild et al., 2001). The investigation of other kinases targeting MDM2 may further shed light on MDM2-p53 function.

It is equally important to consider MDM2 dephosphorylation mechanisms and impact on p53 regulation under cellular stress. Wild type p53-induced phosphatase 1 (Wip/PPM1D) can dephosphorylate MDM2 at serine 395, the same site that is phosphorylated by ATM, to inhibit MDM2 autophosphorylation and to stabilize MDM2 protein (Lu et al., 2007). Activation of p53 induces cyclin G1 transcription after DNA damage. Cyclin G1 interacts with a subunit of protein phosphatase 2A subunit (PP2A) to dephosphorylate MDM2 at tyrosine 216 site, promoting the ARF/MDM2 complex formation and p53 degradation (Kimura and Nojima, 2002; Meek and Knippschild, 2003). In another study, Cyclin G1/PP2A dephosphorylated MDM2 at both tyrosine 216 and serine 166 to activate it and to negatively regulate p53. However, the downstream functions of cyclin G1-p53 are complicated under various cellular stress. Exploring these issues should lead to a further understanding of p53-MDM2 regulation network (Okamoto et al., 2002).

#### Ubiquitination

During the ubiquitin conjugation modification process, the E3 ligase interacts specifically with target proteins to transfer ubiquitin. As a ubiquitin E3 ligase, MDM2 not only ubiquitinates p53 to guide its proteasomal degradation but also exhibits auto-ubiquitination function *via* C terminal RING finger domain (Fang et al., 2000; Honda and Yasuda, 2000). Multiple monoubiquitin sites exist in p53 which requires MDM2 isomerization (Lai et al., 2001). MDM2 auto-ubiquitination facilitates E2-conjugating enzymes accumulation and increased ubiquitination activity of MDM2 substrates, nevertheless the auto-ubiquitination is not required for MDM2 degradation (Itahana et al., 2007; Ranaweera and Yang, 2013). The choice between auto-ubiquitination and substrate-ubiquitination is regulated by other MDM2 posttranslational modifications including phosphorylation and SUMOylation (Meek and Knippschild, 2003). Other proteins are also found in the regulation of MDM2 ubiquitination. Transcription factor Yin Yang 1 (YY1) promotes p53 ubiquitination and degradation, which relays on enhanced p53-MDM2 interaction but not on its transcriptional activity (Sui et al., 2004).

#### SUMOylation

SUMO is a small ubiquitin-related modifier that modulates downstream gene expression in a ubiquitination-like pathway to influence protein stability, localization, DNA binding, and activation (Gill, 2003). Sumo conjugating enzyme Ubc9 mediated SUMOylation shows different consequences according to its substrates and the type of DNA damage. Residues 40–59 on MDM2 are responsible for Ubc9-MDM2 interaction and MDM2 SUMOylation, which is decreased following UV treatment (Buschmann et al., 2001). In response to radiation, the SUMOylation of MDM2 decreased in a dose-and time-dependent manner to enhance MDM2 auto-ubiquitination and to abrogate substrate-ubiquitination, resulting in increased p53 level (Buschmann et al., 2000). SUMOylation of MDM2 modulates MDM2 E3 ligase activity. SUMO-specific protease 2 (SENP2) removes SUMO modification from MDM2 and decrease p53 level (Chiu et al., 2008).

### Regulation of MDM2 Stabilization

MDM2 exhibits auto-ubiquitination function to promote protein degradation by ubiquitin-proteasome pathway in response to cellular stress or DNA damage (Chang et al., 1998). The half-life of MDM2 is short and the ubiquitin-related regulation balance shifts to MDM2 auto-ubiquitination and degradation to relieve p53 inhibition after exposure to these stressors (Stommel and Wahl, 2004). The histone acetyltransferase (HAT) p300-CBP-associated factor (PCAF) was reported to control MDM2 stability and p53 function through its ubiquitination activity (Linares et al., 2007).

### Regulation of MDM2 Localization

The structural property of MDM2 enables it to shuttle between the nucleus and the cytoplasm. Subcellular location of MDM2 is important for regulating p53 transcriptional activity and degradation. In addition to the nuclear entry regulated by MDM2 phosphorylation, MDM2 mediated p53 nuclear export is also required for p53 degradation. MDM2 overexpression promotes p53 cytoplasmic location and this function relies on the RING finger domain in MDM2 and the NES signal in p53 (Boyd et al., 2000; Geyer et al., 2000). By contrast, the export protein CRM1 mediated p53 nuclear export did not cause p53 degradation indicating that the export of p53 is not essential for its degradation (Lohrum et al., 2001). The translocation of MDM2 into nucleus through ARF is needed for MDM2-mediated p53 response (Weber et al., 1999). Another tumor suppressor promyelocytic leukemia (PML) interacts with MDM2 and promotes p53 activity by enhancing the accumulation of p53 at PML nuclear bodies (Wei et al., 2003). Following DNA damage, PML localizes at nucleoli and sequesters MDM2 nucleolar localization to increase p53 stability (Bernardi et al., 2004).

## MDM2 in Cancer Development

The overexpression of MDM2 inhibits p53 activity. Highly expressed MDM2 attenuates p53 stress response and promotes cancer progression. Study in sarcomas identified MDM2 gene amplification together with a decreased growth control by p53 (Oliner et al., 1992). MDM2 overexpression was also found in head and neck squamous carcinomas, indicating its contribution in cancer development (Valentin-Vega et al., 2007). An efficient strategy would be to target MDM2 by improving p53 activity in some cancer types (Vazquez et al., 2008). Since MDM2 functions in p53 dependent and independent manner, studies of the roles of MDM2 in cancer development need to be considered in both situations.

### P53 Dependent Oncogenic Function of MDM2

**Figure 4:**
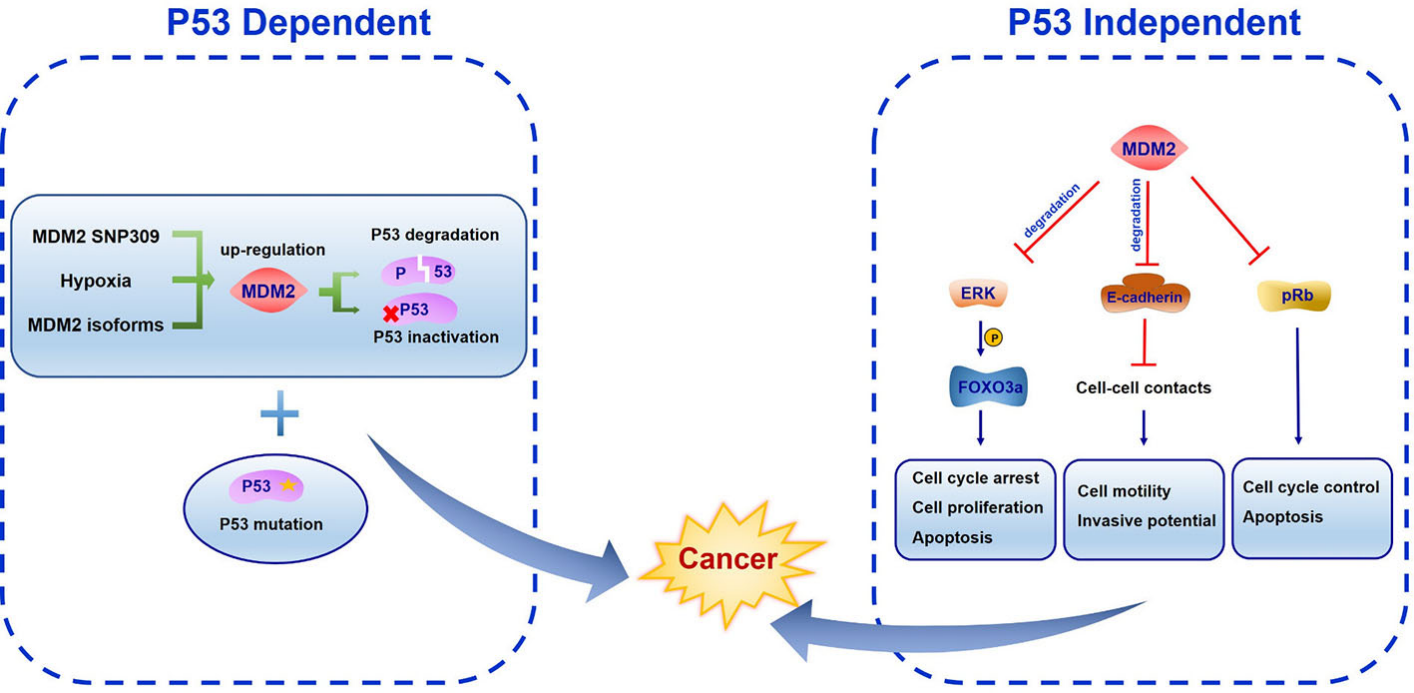
The function of mouse double minute 2 (MDM2) in cancer development. In p53 dependent manner, MDM2 SNP309, hypoxia microenvironment and alternative splice forms all induce MDM2 up-regulation, leading to p53 degradation or inactivation. Both lesions cooperation with other oncogenes like MDM2 overexpression and loss of tumor suppressor genes like p53 can promote cancer development. In p53 independent manner, FOXO3a is phosphorylated by ERK, becoming the ubiquitination target of MDM2. MDM2 binds E-cadherin to facilitate its ubiquitination and degradation. Meanwhile, MDM2 inhibits pRb function.

The most important oncogenic function of MDM2 comes from its binding with transcriptional activation domain and abrogating the antiproliferation effects of tumor suppressor protein p53 (Haupt et al., 1997). MDM2 SNP309 is associated with high risk of cancer development, largely because of increased MDM2 protein level and inhibition of p53 activation (Bond and Levine, 2007). As a common feature of solid tumors, the hypoxia microenvironment is important in tumor growth, progression and metastatic potential. Hypoxia was reported to induce MDM2 up-regulation leading to a decreased p53 function (Zhang and Hill, 2004). Alternative splice forms of MDM2 have been found in various human cancers, indicating diversified regulatory mode of MDM2 and its transcriptional variants (Jeyaraj et al., 2009). Both MDM2 overexpression and/or p53 mutation are exhibited in human cancers (**Figure 4**). Over half of human cancers showed deficient or mutant p53 protein whereas the remaining harbor other kinds of alterations like MDM2 overexpression (Senturk and Manfredi, 2012). Mice homozygous for p53 null allele developed spontaneous tumors with high probability, and most of the tumors were lymphomas (Donehower et al., 1992; Jacks et al., 1994; Purdie et al., 1994). MDM2 plays an essential role in p53 regulation *in vivo*. The evidence came from MDM2 single knockout and MDM2/p53 double knockout mice. It was found that MDM2 loss triggered embryonic lethality in mice. In contrast, mice deficient in both MDM2 and p53 were rescued from this phenotype leading to normal development and survival. These results suggest the function of MDM2 in negative regulating p53 activity (Jones et al., 1995; Montes et al., 1995)

The oncogenic feature of MDM2 only manifests in a background of pre-existing genetic lesions in cell-based studies. Because on the short half-life of MDM2 and the toxic cellular response to MDM2 overexpression, it is difficult to increase MDM2 expression to induce p53 inactivity and trigger transformation or tumorigenesis (Wade et al., 2013). Both lesions cooperate with other oncogenes and loss of tumor suppressor genes can promote tumorigenesis (Wade et al., 2013).

### P53 Independent Oncogenic Function of MDM2

The p53 independent oncogenic function of MDM2 was identified in MDM2 transgenic mice in which mammary gland were prone to spontaneous cancer formation both in the presence or absence of p53 (Lundgren et al., 1997; Jones et al., 1998). One of the underlying mechanisms of this effect may be the MDM2-NBS1 interaction which leads to inhibition of DNA repair and increased transformation efficiency (Bouska et al., 2008). Several MDM2 spliced variants have been found in different cancer types which are absent in normal tissues. All the variants can transform NIH3T3 cells. Moreover, some of them were unable to bind p53 but were still expressed in late-stage and high-grade ovarian and bladder cancer, suggesting a p53-independent oncogenic function (Sigalas et al., 1996). Considering the E3 ligase activity of MDM2, it is reasonable to assume that MDM2 ubiquitinates other targets involved in proliferation and tumorigenesis. The RAS–RAF–MEK–ERK–MAP kinase pathway is the most characterized signal transduction pathway regulating cell cycle arrest, cell proliferation, differentiation, and apoptosis. A wide range of mutations in these genes have been found in human cancers (Davies et al., 2002). ERK was reported to interact with and phosphorylate transcription factor FOXO3a at serine 294, 344, and 425. Phosphorylated FOXO3a is a ubiquitination target of MDM2 and is degraded by MDM2 mediated proteasome pathway in response to oncogenic growth factor signals (Yang et al., 2008). Southern blot and PCR methods have identified more frequent MDM2 gene amplification in metastatic or recurrent osteosarcomas than the corresponding primary cancer (Ladanyi et al., 1993). This can be attributed to E-cadherin, an epithelial-to-mesenchymal transition associated cell-adhesion protein which interacts with MDM2 to facilitate its ubiquitination and degradation (Yang et al., 2006). Paradoxically, Slug (SNAI2), which belongs to Snail transcriptional repressor family, can be downregulated by MDM2 to enhance E-cadherin expression and repress cancer invasiveness (Wang et al., 2009). In addition, enhanced MDM2 expression correlates with increased vascular endothelial growth factor (VEGF) expression which may facilitate vascularity, metastasis, and tumor growth (Takahashi et al., 1995; Zietz et al., 1998). Other than p53, another tumor suppressor gene retinoblastoma protein (pRb) is frequently mutated in human cancers. MDM2 was identified to interact with pRb directly to inhibit its cell cycle and apoptosis regulatory function (Xiao et al., 1995).

## MDM2 as a Target for Cancer Therapy

The regulation functions of MDM2 on p53 focus on ubiquitinating p53 through the E3 ligase activity, blocking the binding of p53 to its target transcription sites and exporting p53 from nucleus, all suggests the necessity to design inhibitors targeting the interaction sites between MDM2 and p53. Considering the deficiency of single target drugs in therapeutic effect maintenance over time as well as the conduciveness to activate alternative signaling pathways facilitating drug resistance, dual or multi-targeting MDM2 inhibitors are emerging. Here, we review inhibitors which have been successfully developed for the clinical trials (**Table 1**).

**Table 1:** Overview of small-molecules targeting mouse double minute 2 (MDM2) in clinical trials.

Drug	Phases	NCT Number	Status	Conditions
RG7211	Phase 1	NCT00559533	Completed	Neoplasms
RG7211	Phase 1	NCT00623870	Completed	Hematologic neoplasms
RG7211	Phase 1	NCT01143740	Completed	Sarcoma
RG7211	Phase 1	NCT01164033	Completed	Neoplasms
RG7211	Phase 1	NCT01605526	Completed	Sarcoma
RG7211	Phase 1	NCT01635296	Completed	Myelogenous leukemia, acute
RG7211	Phase 1	NCT01677780	Completed	Myelogenous leukemia, chronic, neoplasms, myelogenous leukemia, acute
RG7388	Phase 1	NCT01462175	Completed	Neoplasms
RG7388	Phase 1	NCT01773408	Completed	Myelogenous leukemia, acute
RG7388	Phase 1	NCT01901172	Completed	Neoplasms
RG7388	Phase 3	NCT02545283	Recruiting	Leukemia, myeloid, acute
RG7388	Phase 1|phase 2	NCT02633059	Recruiting	Loss of chromosome 17p|recurrent plasma cell myeloma
RG7388	Phase 1|phase 2	NCT02670044	Recruiting	Leukemia, myeloid, acute
RG7388	Phase 1|phase 2	NCT03135262	Active, not recruiting	Follicular lymphoma|lymphoma, large B-cell, diffuse
RG7388	Phase 1|phase 2	NCT03158389	Recruiting	Glioblastoma, adult
RG7388	Phase 1|phase 2	NCT03337698	Recruiting	Carcinoma, non-small-cell lung
RG7388	Phase 1	NCT03362723	Completed	Solid tumors
RG7388	Phase 1|phase 2	NCT03555149	Recruiting	Colorectal cancer
RG7388	Phase 1|phase 2	NCT03566485	Active, not recruiting	Stage III breast cancer|stage IIIA breast cancer|stage IIIB breast cancer|stage IIIC breast cancer|stage IV breast cancer|estrogen receptor-positive|HER2/Neu negative
RG7388	Phase 1|phase 2	NCT04029688	Recruiting	AML|ALL|neuroblastoma|solid tumors
RG7775	Phase 1	NCT02098967	Completed	Neoplasms, myelogenous leukemia, acute
SAR405838	Phase 1	NCT01636479	Completed	Neoplasm malignant
SAR405838	Phase 1	NCT01985191	Completed	Neoplasm malignant
HDM201	Phase 1	NCT02143635	Active, not recruiting	Advanced solid and hematological TP53wt tumors
HDM201	Phase 1|phase 2	NCT02343172	Active, not recruiting	Liposarcoma
HDM201	Phase 1	NCT02601378	Active, not recruiting	Uveal melanoma
HDM201	Phase 1	NCT02780128	Recruiting	Neuroblastoma|cancer
HDM201	Phase 1	NCT02890069	Recruiting	Colorectal cancer, non-small cell lung carcinoma (adenocarcinoma), triple negative breast cancer, renal cell carcinoma
HDM201	Phase 1	NCT03714958	Recruiting	Colorectal cancer|advanced cancer|metastatic cancer
HDM201	Phase 1|phase 2	NCT03760445	Recruiting	Leukemia, myeloid, acute
HDM201	Phase 1	NCT03940352	Recruiting	AML|high-risk MDS
HDM201	Phase 2	NCT04116541	Not yet recruiting	Malignant solid tumor
APG-115	Phase 1	NCT02935907	Recruiting	Patients with advanced solid tumor or lymphoma
APG-115	Phase 1|phase 2	NCT03611868	Recruiting	Unresectable or metastatic melanoma or advanced solid tumors
APG-115	Phase 1|phase 2	NCT03781986	Not yet recruiting	Malignant salivary gland cancer
AMG-232	Phase 1	NCT01723020	Completed	Advanced malignancy|advanced solid tumors|cancer|oncology|oncology patients|tumors|glioblastoma|multiple myeloma
AMG-232	Phase 1	NCT02016729	Completed	Advanced Malignancy|cancer|oncology|oncology Patients|AML
AMG-232	Phase 1|phase 2	NCT02110355	Completed	Advanced malignancy|advanced solid tumors|cancer|oncology|oncology patients|tumors|melanoma
AMG-232	Phase 1	NCT03031730	Recruiting	Hypercalcemia|plasmacytoma|recurrent plasma cell myeloma|refractory plasma cell myeloma
AMG-232	Phase 1	NCT03041688	Recruiting	AML|recurrent AML|refractory AML|secondary AML
AMG-232	Phase 1	NCT03107780	Recruiting	Glioblastoma|gliosarcoma|recurrent glioblastoma
AMG-232	Phase 1	NCT03217266	Recruiting	Soft tissue sarcoma
AMG-232	Phase 2	NCT03662126	Recruiting	PMF|Post-PV-MF|Post-ET-MF
AMG-232	Phase 2	NCT03669965	Recruiting	Polycythemia vera
AMG-232	Phase 2	NCT03787602	Recruiting	Merkel cell carcinoma
AMG-232	Phase 1|phase 2	NCT04113616	Recruiting	AML|AML, secondary to MPN
AMG-232	Phase 1	NCT04190550	Not yet recruiting	AML|AML arising from previous MDS
MK-8242	Phase 1	NCT01451437	Terminated	AML
MK-8242	Phase 1	NCT01463696	Terminated	Solid tumors

AML, acute myeloid leukemia; MDS, myelodysplastic syndrome; PMF, primary myelofibrosis; Post-PV-MF, post-polycythemia vera MF; Post-ET-MF, post-essential thrombocythemia MF; MPN, myeloproliferative neoplasms.

### Inhibitors Targeting MDM2-p53 in Clinical Trials

Nutlins was a series of cis-imidazoline analogs identified to bind MDM2 in the p53-binding pocket, leading to cell cycle arrest and apoptosis in cancer cells, as well as growth inhibition of human tumor xenografts in nude mice (Vassilev et al., 2004). Several inhibitors targeting MDM2-p53 such as RG7112, RG7388, RG7775, SAR405838, HDM201, APG-115, AMG-232, and MK-8242 have recently been developed to treat human cancers with clinical trials. However, more clinical data is required to verify if these inhibitors can effectively block MDM2 or activate p53 and further improve the clinical efficacy in patients.

#### RG7112

RG7112 was the first small-molecule MDM2 inhibitor to enter human clinical trials and which was derived from structural modification of Nutlin-3a (Liu et al., 2019). RG7112 was designed to target MDM2 in p53-binding pocket (Vu et al., 2013) and restored p53 activity inducing robust p21 expression and apoptosis in p53 wild-type glioblastomas cell (Verreault et al., 2016). So far, seven clinical studies on RG7112 have been completed (http://www.clinicaltrials.gov/; NCT01677780, NCT01605526, NCT01143740, NCT01164033, NCT00559533, NCT00623870, NCT01677780). Study of NP25299 (NCT01164033) was an open-label, randomized, cross-over study in patients with solid tumors. It evaluated the effects of food on the pharmacokinetics of single oral doses of RG7112. This study included two parts: the first one comprised an initial single-dose, while the other comprised four different treatment schedules of increased doses. The results indicated that RG7112 was generally well tolerated with GI toxicities, the most common AEs, making it treatable with anti-emetics (Patnaik et al., 2015).

#### RG7388

RG7388, a second-generation Nutlin, was developed to improve the potency and toxicity profile of earlier Nutlin. RG7388 induced p21 expression and effective cell cycle arrest in three cell lines MCF-7, U-2OS and SJSA-1, which proved the strong activation of p53 (Skalniak et al., 2018). RG7388 is currently undergoing several clinical examinations, including the only III clinical trial of MDM2 inhibitor (MIRROS/NCT02545283). The results of phase I clinical trial showed that RG7388 improved clinical outcomes by modulating p53 activity in AML patients with high levels of MDM2 expression (Reis et al., 2016). MIRROS is a randomized phase III clinical trial to evaluate the efficacy of RG7388 combined with cytarabine in the treatment of recurrent and refractory acute myeloid leukemia (AML). As of April 2019, the study has recruited approximately 90% of patient population and is still ongoing. If 80% of deaths are observed in p53-WT population of this study, an interim efficacy analysis can be obtained by 2020. MIRROS may obtain the first phase III clinical trial data of MDM2 inhibitors and provide a new treatment option for patients with AML (Montesinos et al., 2020).

#### RG7775

RG7775 is an inactive pegylated prodrug of AP (idasanutlin), which cleaves the pegylated tail of esterases in the blood. AP is a potent and selective inhibitor of p53-MDM2 interaction to activate p53 pathway and associates with cell-cycle arrest and/or apoptosis. In a preclinical trial, intravenous (IV) RG7775 (RO6839921) showed anti-tumor effects in osteosarcoma and AML in immunocompromised mice model (Chen et al., 2019). In a phase I study (NCT02098967), RG7775 was investigated for its safety, tolerability, and pharmacokinetics in patients with advanced malignancies (Abdul et al., 2019). The result showed that RG7775 had a safety profile comparable to oral idasanutlin.

#### SAR405838

SAR405838 is an oral selective spirooxindole small molecule derivative antagonist of MDM2, which targets MDM2-p53 interaction (Wang et al., 2014). In the treatment of dedifferentiated liposarcoma cells, SAR405838 effectively stabilized p53, activated p53 pathway, block cell proliferation, promoted cell-cycle arrest and induced apoptosis (Bill et al., 2016). SAR405838 has been used in two clinical trials in cancer patients (NCT01636479, NCT01985191). Study of TED12318 (NCT01636479) was a phase I, open-label, dose-ranging, dose escalating, safety study administered orally in adult patients with advanced solid tumor. In this trial, 74 patients were treated with SAR405838 which showed best response in 56% patients with a 32% 3-month progression free rate. This study indicated that SAR405838 had an acceptable safety profile in patients with advanced solid tumors (de Jonge et al., 2017). Another clinical trial on SAR405838 was the study of TCD13388 (NCT01985191), which analyzed safety and efficacy of SAR405838 combined with pimasertib in cancer patients. In this study, 26 patients with locally advanced or metastatic solid tumors, who were documented to have wild-type p53 and RAS or RAF mutations, were enrolled in this study. The aim of this study was to explore maximum tolerated dose (MTD). Patient response was observed with SAR405838 at 200 or 300 mg QD plus pimasertib 60 mg QD or 45 mg BID. The most frequently occurring adverse events observed were diarrhoea (81%), blood creatine phosphokinase (77%), nausea (62%) and vomiting (62%). This study indicated that the safety profile of SAR405838 combined with pimasertib was consistent with the safety profiles of both the drugs (de Weger et al., 2019).

#### HDM201

HDM201 is a potent and selective small molecule that inhibits the interaction between MDM2 and p53, leading to tumor regression in preclinical models with both low and high dose regimen (Seipel et al., 2018). HDM201 had a specific and effective killing effect on p53 wild-type cells with positive-ITD when used in combination with midotaline (Seipel et al., 2018). HDM201 has been used in clinal trial (NCT02143635). NCT02143635 determined and evaluated a safe and tolerated dose of HDM201 in patients with advanced tumors with wild type p53. At the time of data cut-off (April 1, 2016), 74 patients received HDM201 (Reg 1 with 38 patients and Reg 2 with 36 patients still receiving treatment). The results showed that the common grade 3/4 adverse events (AEs) in both regimens (Reg 1 and Reg 2) were anemia (8%; 17%), neutropenia (26%; 14%), and thrombocytopenia (24%; 28%). Preliminary data indicated that hematological toxicity was delayed and dependent on regimen and that the Reg 1 regimen allows for higher cumulative dose (Jeay et al., 2018).

#### APG-115

APG-115 is a novel, orally active small-molecule MDM2 inhibitor. APG-115 restores p53 expression after binding with MDM2 and activates p53 mediated apoptosis in tumor cells with wild-type p53 (Aguilar et al., 2017). APG-115 has been used in clinical trials for treating solid tumor (NCT02935907), metastatic melanoma (NCT03611868), and salivary gland carcinoma (NCT03781986). Study NCT02935907 was a phase I study of the safety, pharmacokinetic and pharmacodynamic properties of orally administered APG-115 in patients with advanced solid tumors or lymphomas. Different dose levels (Including 10 mg, 20 mg, 50 mg, 100 mg, 200 mg and 300 mg) were tested in this study. The result showed the optimum dose of APG-115 to be 100 mg with no dose-limiting toxicities (Tolcher et al., 2019). In recent studies, APG-115 mediated the anti-tumor immunity of tumor microenvironment (TME). APG-115 activated p53 and p21 on bone marrow-derived macrophages *in vitro*, and reduced the number of immunosuppressive M2 macrophages by down-regulating c-Myc and c-Maf. In addition, APG-115 showed costimulatory activity in T cells and increased the expression of PD-L1 in tumor cells. This evidence suggests the combination of APG and immunotherapy may be a new anti-tumor regimen (Fang et al., 2019).

#### AMG 232

AMG 232 is an investigational oral, selective MDM2 inhibitor that restores p53 tumor suppression by blocking MDM2-p53 interaction (Sun et al., 2014). The activity of AMG 232 and its effect on p53 signal were characterized in several preclinical tumor models. AMG 232 bind MDM2, strongly induced p53 activity, lead to cell cycle arrest and inhibit tumor cell proliferation (Canon et al., 2015). Several clinical trials of the AMG 232 such as NCT01723020, NCT02016729, NCT02110355, NCT03031730, NCT03041688, NCT03107780, and NCT03217266 have been ongoing to treat human cancers. NCT02016729 was an open-label phase I study that evaluated the safety, pharmacokinetics, and MTD of AMG 232. In this study, AMG 232 was administered in two regimens (arm 1 and arm 2). Patients were treated with AMG 232 at 60, 120, 240, 360, 480, or 960 mg as monotherapy once daily for 7 d every 2 weeks in arm 1 or at 60 mg combined with trametinib at 2 mg in arm 2. The results exhibited common treatment-related AEs included nausea (58%), diarrhea (56%), vomiting (33%), and decreased appetite (25%). However, the MTD of AMG 232 was not reached. Dose escalation was discontinued because of its unacceptable gastrointestinal AEs at higher doses (Erba et al., 2019).

#### MK-8242

MK-8242 is a potent, small-molecule inhibitor which targets MDM2-p53 interaction (Kang et al., 2016). MK-8242 induced tumor regression of various solid tumor types and complete or partial response in most acute lymphoblastic leukemia xenografts (Kang et al., 2016). MK-8242 has been used in two Phase I clinical trials (NCT01451437 and NCT01463696). Study of NCT01451437 was a study of MK-8242 alone and in combination with cytarabine in adult participants with refractory or recurrent AML. In this study MK-8242 was administered at 30–250 mg (p.o;QD) or 120–250 mg (p.o;BID) for 7 d on/7 d off in a 28-d cycle and optimized regimen was administered at 210 or 300 mg (p.o;BID) for 7 on/14 off in 21-d cycle. Twenty-six patients were enrolled in this study, out of which 5 discontinued because of AEs and 7 patients died. This study showed the 7 on/14 off regimen had a more favorable safety profile than the 7 on/7 off regimen (Ravandi et al., 2016). NCT01463696 was aimed at evaluating the safety and pharmacokinetic profile of MK-8242 in patients with advanced solid tumors. In this study, drug dose was escalated to determine the MTD in part 1 and the MTD was confirmed and the recommended Phase 2 dose (RPTD) was established in part 2. Finally, 47 patients were enrolled in this study and treated with MK-8242 at eight level doses that ranged from 60 to 500 mg. The result showed that MK-8242 activated p53 pathway with an acceptable tolerability proﬁle at 400 mg (BID) (Wagner et al., 2017).

### MDM2-Based Multi-Target Inhibitors

Along with the development of MDM2 inhibitors, searching for multi-target inhibitors may be a new research direction. MDM2-based multi-target inhibitors could improve the efficacy and reduce the side effects (de Lera and Ganesan, 2016). Dual inhibitors that have been reported to be co-inhibit with MDM2 include MDM4, NF-κB, histone deacetylases (HDAC), translocator protein (TSPO), XIAP, and integrins (McCormack et al., 2012; Daniele et al., 2016; Gu et al., 2016; Shirai et al., 2016; Giustiniano et al., 2017; Merlino et al., 2018), which achieved excellent *in vitro* and *in vivo* antitumor potency. Considering MDM2 and MDM4 dual inhibitors had been well reviewed in the recent literature (Teveroni et al., 2016; Espadinha et al., 2018), we summarize other dual inhibitors.

#### Dual Inhibitors of MDM2 and NF-κB

Nuclear transcription factor (NF-κB) is a class of key nuclear transcription factors exist in the cytoplasm of almost all types cells in the form of homologous or heterodime. NF-κB family includes five DNA-binding proteins that are usually overexpressed in mammalian cancer cell (Pahl, 1999). Previous studies found that NF-κB inhibit p53 stability by directly up-regulating MDM2 (Dey et al., 2008). The cross-talk between p53 and NF-κB showed that simultaneous activation of p53 and inhibition of NF-κB would provide a synergistic effect on anti-tumor activity. Zhang et al. designed a series of pyrrolo[3,4-c]pyrazole derivatives and synthesized as the first-in-class inhibitors for both p53-MDM2 interaction and NF-κB. These compounds effective targeted p53-MDM2 interaction and inhibit cell growth in A549 xenograft model (Zhuang et al., 2014). In addition, dual-target inhibitors enhanced the anti-tumor effect of radiotherapy in pancreatic cancer (Shirai et al., 2016).

#### Dual Inhibitors of MDM2 and HDAC

Histone deacetylase (HDAC) is a kind of epigenetic enzyme plays an important role in the regulation of tumor suppressor genes (Kouzarides, 2007). In recent years, more HDAC inhibitors have been approved by FDA such as romidepsin and vorinostat (Mann et al., 2007). Most of the inhibitors used in clinic are not effective in the treatment of solid tumors, and they are also limited in the treatment of hematological malignant tumors (Tan et al., 2010). Recent studies showed that HDAC inhibitors cooperated with MDM2 inhibitors to suppress the proliferation of tumor cells (McCormack et al., 2012). Inhibition of HDAC not only lead to the accumulation of activated p53, but also modified the hyperacetylation of p53 to enhance the anti-tumor activity of MDM2 inhibitors (Xu et al., 2007; McCormack et al., 2012). He et al. designed and synthesized the first MDM2/HDAC dual inhibitor according to the known binding model of MDM2 and HDAC inhibitors. These synthetic compounds showed excellent targeted inhibitory effects on HDAC and MDM2 in analysis of biochemical evaluation and structure-activity relationship as well as good anti-tumor activity in the xenograft model of A549 *in vivo* (He et al., 2018). These studies suggest that inhibitors targeting both HDAC and MDM2 may be a new way to treat cancer.

#### Dual Inhibitors of MDM2 and TSPO

The translocation protein (TSPO) is an 18 kDa transmembrane protein, which mainly exists in the outer membrane of mitochondria and constitutes a key part of the mitochondrial permeability transition pore (MPTP) (Austin et al., 2013). MPTP could affect apoptosis by regulating mitochondrial outer membrane permeabilization (MOMP) (Tait and Green, 2010), suggesting TSPO as a useful target to trigger cellular apoptosis (Chelli et al., 2005). Welsch.et al confirmed that multi-target single molecules of TSPO and MDM2 would enhance the antitumor efficacy (Welsch et al., 2010). A 2-phenylindolylglyoxylyl dipeptide was designed to bind TSPO, leading to the disruption of MDM2-p53 interaction, cell-cycle arrest, and apoptosis in human GBM cells (Daniele et al., 2016).

#### Dual Inhibitors of MDM2 and XIAP

XIAP belongs to IAP family, which can down-regulate the mitochondrial apoptosis by inhibiting caspase3, 7, and 9. XIAP is highly expressed in various types of cancer and is related to poor outcome (Tamm et al., 2004; Berezovskaya et al., 2005; Mizutani et al., 2007). The binding between IRES region of XIAP and MDM2 induced translation enhancement of XIAP and stability of MDM2 (Candeias et al., 2008; Liu et al., 2015). Zhou et al. developed a protein-RNA fluorescence polarization (FP) assay for high-throughput screening (HTS) of chemical libraries and identified eight inhibitors blocking the MDM2 protein-XIAP RNA interaction. The compound-induced MDM2 downregulation resulted in inhibition of XIAP expression and in activation of p53, which contributed to cell apoptosis and proliferation inhibition (Gu et al., 2016).

#### Dual Inhibitors of MDM2 and Integrins

Fibronectin is a gene cluster related to highly malignant glioblastoma and includes main receptors α5β1, αvβ6, αvβ8, and αvβ3 integrins (Dechantsreiter et al., 1999; Mas-Moruno et al., 2010). A selective antagonist of integrin receptors perturbed α5β1 function, provoked cell cycle arrest and decreased cell aggressiveness (Maglott et al., 2006). An effective α5β1/αvβ3 integrin binding agent was identified to inhibit MDM2/4 activity and reactivated p53 pathway. In addition, this compound can induce cell cycle arrest, reduce the invasiveness of GBM cells and block the proliferation of p53 wild-type GBM cells (Merlino et al., 2018). The novel class of integrin/MDM2 inhibitors is useful in the subpopulation of patients with glioblastoma expressing functional p53 concomitantly with a high level of α5β1 integrin (Merlino et al., 2018).

## Conclusion

Genotoxic attacks are a huge threat to genomic stability. With the major role of p53 in maintaining genome integrity, it is a big challenge to dissect out diverse regulatory roles of p53. Since the first identification of MDM2 cDNA repeats in transformed mouse 3T3 cells, evidence has increasing supported the functions of MDM2 in regulating cell proliferation, senescence, DNA repair, and apoptosis through p53-dependent and p53-independent pathways. MDM2 forms a large protein interactome to participate in different signal pathways, with the consideration of its changed protein conformations, modulated protein isoforms, tightly regulated post-translational modifications and subcellular localization. MDM2 keeps p53 content to a basal level in normal conditions. In response to stress signals like DNA damage, both MDM2 and p53 undergo diverse post-translational modifications, resulting in p53 stability and activation to regulate target genes transcription. One of the strategies for drug discovery is to improve p53 activity by inhibiting MDM2-p53 interaction. Further studies are needed to clarify the cell fate determination by MDM2-p53 axis after DNA damage and other regulatory pathways of MDM2 protein, especially the diverse isoforms of MDM2 in DNA repair process.

## Author Contributions

All authors substantially contributed to this work. WL and XP designed and performed original draft. WL and XP contributed to figure and table preparation. WL, JL, and CX reviewed and edited the manuscript. All authors have read and agreed to the published version of the manuscript.

## Funding

This study was supported by the National Natural Science Foundation of China (No.81572892, No. 81873048).

## Conflict of Interest

The authors declare that the research was conducted in the absence of any commercial or financial relationships that could be construed as a potential conflict of interest.

## References

[B1] Abdul R. A. Miller W. J. Uy G. L. Blotner S. Young A. M. Higgins B. (2019). A phase 1 study of the MDM2 antagonist RO6839921, a pegylated prodrug of idasanutlin, in patients with advanced solid tumors. Invest. New Drugs. 10.1007/s10637-019-00869-2 31734832

[B2] Aguilar A. Lu J. Liu L. Du D. Bernard D. McEachern D. (2017). Discovery of 4-((3′R,4′S,5′R)-6″-Chloro-4′-(3-chloro-2-fluorophenyl)-1′-ethyl-2″-oxodispiro[ cyclohexane-1,2′-pyrrolidine-3′,3″-indoline]-5′-carboxamido)bicyclo[2.2.2]octane-1-carboxylic Acid (AA-115/APG-115): A Potent and Orally Active Murine Double Minute 2 (MDM2) Inhibitor in Clinical Development. J. Med. Chem. 60 (7), 2819–2839. 10.1021/acs.jmedchem.6b01665 28339198 PMC5394527

[B3] Aguilera A. Garcia-Muse T. (2013). Causes of genome instability. Annu. Rev. Genet. 47, 1–32. 10.1146/annurev-genet-111212-133232 23909437

[B4] Alt J. R. Bouska A. Fernandez M. R. Cerny R. L. Xiao H. Eischen C. M. (2005). Mdm2 binds to Nbs1 at sites of DNA damage and regulates double strand break repair. J. Biol. Chem. 280 (19), 18771–18781. 10.1074/jbc.M413387200 15734743

[B5] Appella E. Anderson C. W. (2001). Post-translational modifications and activation of p53 by genotoxic stresses. Eur. J. Biochem. 268 (10), 2764–2772. 10.1046/j.1432-1327.2001.02225.x 11358490

[B6] Arva N. C. Gopen T. R. Talbott K. E. Campbell L. E. Chicas A. White D. E. (2005). A chromatin-associated and transcriptionally inactive p53-Mdm2 complex occurs in mdm2 SNP309 homozygous cells. J. Biol. Chem. 280 (29), 26776–26787. 10.1074/jbc.M505203200 15908423

[B7] Asahara H. Li Y. Fuss J. Haines D. S. Vlatkovic N. Boyd M. T. (2003). Stimulation of human DNA polymerase epsilon by MDM2. Nucleic Acids Res. 31 (9), 2451–2459. 10.1093/nar/gkg342 12711691 PMC154228

[B8] Austin C. J. Kahlert J. Kassiou M. Rendina L. M. (2013). The translocator protein (TSPO): a novel target for cancer chemotherapy. Int. J. Biochem. Cell Biol. 45 (7), 1212–1216. 10.1016/j.biocel.2013.03.004 23518318

[B9] Barak Y. Gottlieb E. Juven-Gershon T. Oren M. (1994). Regulation of mdm2 expression by p53: alternative promoters produce transcripts with nonidentical translation potential. Genes Dev. 8 (15), 1739–1749. 10.1101/gad.8.15.1739 7958853

[B10] Baskaran R. Wood L. D. Whitaker L. L. Canman C. E. Morgan S. E. Xu Y. (1997). Ataxia telangiectasia mutant protein activates c-Abl tyrosine kinase in response to ionizing radiation. Nature 387 (6632), 516–519. 10.1038/387516a0 9168116

[B11] Berezovskaya O. Schimmer A. D. Glinskii A. B. Pinilla C. Hoffman R. M. Reed J. C. (2005). Increased expression of apoptosis inhibitor protein XIAP contributes to anoikis resistance of circulating human prostate cancer metastasis precursor cells. Cancer Res. 65 (6), 2378–2386. 10.1158/0008-5472.CAN-04-2649 15781653

[B12] Bernardi R. Scaglioni P. P. Bergmann S. Horn H. F. Vousden K. H. Pandolfi P. P. (2004). PML regulates p53 stability by sequestering Mdm2 to the nucleolus. Nat. Cell Biol. 6 (7), 665–672. 10.1038/ncb1147 15195100

[B13] Bill K. L. Garnett J. Meaux I. Ma X. Creighton C. J. Bolshakov S. (2016). SAR405838: A Novel and Potent Inhibitor of the MDM2:p53 Axis for the Treatment of Dedifferentiated Liposarcoma. Clin. Cancer Res. 22 (5), 1150–1160. 10.1158/1078-0432.CCR-15-1522 26475335 PMC4775372

[B14] Blattner C. Hay T. Meek D. W. Lane D. P. (2002). Hypophosphorylation of Mdm2 augments p53 stability. Mol. Cell Biol. 22 (17), 6170–6182. 10.1128/mcb.22.17.6170-6182.2002 12167711 PMC134018

[B15] Bond G. L. Levine A. J. (2007). A single nucleotide polymorphism in the p53 pathway interacts with gender, environmental stresses and tumor genetics to influence cancer in humans. Oncogene 26 (9), 1317–1323. 10.1038/sj.onc.1210199 17322917

[B16] Bond G. L. Hu W. Bond E. E. Robins H. Lutzker S. G. Arva N. C. (2004). A single nucleotide polymorphism in the MDM2 promoter attenuates the p53 tumor suppressor pathway and accelerates tumor formation in humans. Cell 119 (5), 591–602. 10.1016/j.cell.2004.11.022 15550242

[B17] Bosch-Presegue L. Raurell-Vila H. Marazuela-Duque A. Kane-Goldsmith N. Valle A. Oliver J. (2011). Stabilization of Suv39H1 by SirT1 is part of oxidative stress response and ensures genome protection. Mol. Cell 42 (2), 210–223. 10.1016/j.molcel.2011.02.034 21504832

[B18] Bottger A. Bottger V. Sparks A. Liu W. L. Howard S. F. Lane D. P. (1997). Design of a synthetic Mdm2-binding mini protein that activates the p53 response in vivo. Curr. Biol. 7 (11), 860–869. 10.1016/s0960-9822(06)00374-5 9382809

[B19] Bouska A. Eischen C. M. (2009). Mdm2 affects genome stability independent of p53. Cancer Res. 69 (5), 1697–1701. 10.1158/0008-5472.CAN-08-3732 19244108

[B20] Bouska A. Lushnikova T. Plaza S. Eischen C. M. (2008). Mdm2 promotes genetic instability and transformation independent of p53. Mol. Cell Biol. 28 (15), 4862–4874. 10.1128/MCB.01584-07 18541670 PMC2493369

[B21] Boyd S. D. Tsai K. Y. Jacks T. (2000). An intact HDM2 RING-finger domain is required for nuclear exclusion of p53. Nat. Cell Biol. 2 (9), 563–568. 10.1038/35023500 10980695

[B22] Brown L. McCarthy N. (1997). DNA repair. A sense-abl response? Nature 387 (6632), 450–451. 10.1038/387450a0 9168102

[B23] Bunting S. F. Nussenzweig A. (2013). End-joining, translocations and cancer. Nat. Rev. Cancer 13 (7), 443–454. 10.1038/nrc3537 23760025 PMC5724777

[B24] Buschmann T. Fuchs S. Y. Lee C. G. Pan Z. Q. Ronai Z. (2000). SUMO-1 modification of Mdm2 prevents its self-ubiquitination and increases Mdm2 ability to ubiquitinate p53. Cell 101 (7), 753–762. 10.1016/s0092-8674(00)80887-9 10892746

[B25] Buschmann T. Lerner D. Lee C. G. Ronai Z. (2001). The Mdm-2 amino terminus is required for Mdm2 binding and SUMO-1 conjugation by the E2 SUMO-1 conjugating enzyme Ubc9. J. Biol. Chem. 276 (44), 40389–40395. 10.1074/jbc.M103786200 11384992

[B26] Candeias M. M. Malbert-Colas L. Powell D. J. Daskalogianni C. Maslon M. M. Naski N. (2008). P53 mRNA controls p53 activity by managing Mdm2 functions. Nat. Cell Biol. 10 (9), 1098–1105. 10.1038/ncb1770 19160491

[B27] Canon J. Osgood T. Olson S. H. Saiki A. Y. Robertson R. Yu D. (2015). The MDM2 Inhibitor AMG 232 Demonstrates Robust Antitumor Efficacy and Potentiates the Activity of p53-Inducing Cytotoxic Agents. Mol. Cancer Ther. 14 (3), 649–658. 10.1158/1535-7163.MCT-14-0710 25567130

[B28] Cao Z. Xue J. Cheng Y. Wang J. Liu Y. Li H. (2019). MDM2 promotes genome instability by ubiquitinating the transcription factor HBP1. Oncogene 38 (24), 4835–4855. 10.1038/s41388-019-0761-2 30816344 PMC6756050

[B29] Carroll P. E. Okuda M. Horn H. F. Biddinger P. Stambrook P. J. Gleich L. L. (1999). Centrosome hyperamplification in human cancer: chromosome instability induced by p53 mutation and/or Mdm2 overexpression. Oncogene 18 (11), 1935–1944. 10.1038/sj.onc.1202515 10208415

[B30] Chang Y. C. Lee Y. S. Tejima T. Tanaka K. Omura S. Heintz N. H. (1998). mdm2 and bax, downstream mediators of the p53 response, are degraded by the ubiquitin-proteasome pathway. Cell Growth Differ. 9 (1), 79–84. 9438391

[B31] Chelli B. Rossi L. Da P. E. Costa B. Spinetti F. Rechichi M. (2005). PIGA (N,N-Di-n-butyl-5-chloro-2-(4-chlorophenyl)indol-3-ylglyoxylamide), a new mitochondrial benzodiazepine-receptor ligand, induces apoptosis in C6 glioma cells. Chembiochem 6 (6), 1082–1088. 10.1002/cbic.200400350 15883977

[B32] Chen L. Pastorino F. Berry P. Bonner J. Kirk C. Wood K. M. (2019). Preclinical evaluation of the first intravenous small molecule MDM2 antagonist alone and in combination with temozolomide in neuroblastoma. Int. J. Cancer 144 (12), 3146–3159. 10.1002/ijc.32058 30536898 PMC6491995

[B33] Cheng Q. Chen L. Li Z. Lane W. S. Chen J. (2009). ATM activates p53 by regulating MDM2 oligomerization and E3 processivity. EMBO J. 28 (24), 3857–3867. 10.1038/emboj.2009.294 19816404 PMC2797053

[B34] Cheng Q. Cross B. Li B. Chen L. Li Z. Chen J. (2011). Regulation of MDM2 E3 ligase activity by phosphorylation after DNA damage. Mol. Cell Biol. 31 (24), 4951–4963. 10.1128/MCB.05553-11 21986495 PMC3233024

[B35] Chiu S. Y. Asai N. Costantini F. Hsu W. (2008). SUMO-specific protease 2 is essential for modulating p53-Mdm2 in development of trophoblast stem cell niches and lineages. PloS Biol. 6 (12), e310. 10.1371/journal.pbio.0060310 19090619 PMC2602722

[B36] Ciccia A. Elledge S. J. (2010). The DNA damage response: making it safe to play with knives. Mol. Cell 40 (2), 179–204. 10.1016/j.molcel.2010.09.019 20965415 PMC2988877

[B37] Cross B. Chen L. Cheng Q. Li B. Yuan Z. M. Chen J. (2011). Inhibition of p53 DNA binding function by the MDM2 protein acidic domain. J. Biol. Chem. 286 (18), 16018–16029. 10.1074/jbc.M111.228981 21454483 PMC3091211

[B38] D’Amours D. Jackson S. P. (2002). The Mre11 complex: at the crossroads of dna repair and checkpoint signalling. Nat. Rev. Mol. Cell Biol. 3 (5), 317–327. 10.1038/nrm805 11988766

[B39] Daniele S. La Pietra V. Barresi E. Di Maro S. Da P. E. Robello M. (2016). Lead Optimization of 2-Phenylindolylglyoxylyldipeptide Murine Double Minute (MDM)2/Translocator Protein (TSPO) Dual Inhibitors for the Treatment of Gliomas. J. Med. Chem. 59 (10), 4526–4538. 10.1021/acs.jmedchem.5b01767 27050782

[B40] Davies H. Bignell G. R. Cox C. Stephens P. Edkins S. Clegg S. (2002). Mutations of the BRAF gene in human cancer. Nature 417 (6892), 949–954. 10.1038/nature00766 12068308

[B41] de Jonge M. de Weger V. A. Dickson M. A. Langenberg M. Le Cesne A. Wagner A. J. (2017). A phase I study of SAR405838, a novel human double minute 2 (HDM2) antagonist, in patients with solid tumours. Eur. J. Cancer 76, 144–151. 10.1016/j.ejca.2017.02.005 28324749

[B42] de Lera A. R. Ganesan A. (2016). Epigenetic polypharmacology: from combination therapy to multitargeted drugs. Clin. Epigenet. 8, 105. 10.1186/s13148-016-0271-9 PMC5062873 27752293

[B43] de Toledo S. M. Azzam E. I. Dahlberg W. K. Gooding T. B. Little J. B. (2000). ATM complexes with HDM2 and promotes its rapid phosphorylation in a p53-independent manner in normal and tumor human cells exposed to ionizing radiation. Oncogene 19 (54), 6185–6193. 10.1038/sj.onc.1204020 11175332

[B44] de Weger V. A. de Jonge M. Langenberg M. Schellens J. Lolkema M. Varga A. (2019). A phase I study of the HDM2 antagonist SAR405838 combined with the MEK inhibitor pimasertib in patients with advanced solid tumours. Br. J. Cancer 120 (3), 286–293. 10.1038/s41416-018-0355-8 30585255 PMC6354023

[B45] Dechantsreiter M. A. Planker E. Matha B. Lohof E. Holzemann G. Jonczyk A. (1999). N-Methylated cyclic RGD peptides as highly active and selective alpha(V)beta(3) integrin antagonists. J. Med. Chem. 42 (16), 3033–3040. 10.1021/jm970832g 10447947

[B46] Dey A. Tergaonkar V. Lane D. P. (2008). Double-edged swords as cancer therapeutics: simultaneously targeting p53 and NF-kappaB pathways. Nat. Rev. Drug Discovery 7 (12), 1031–1040. 10.1038/nrd2759 19043452

[B47] Dias S. S. Milne D. M. Meek D. W. (2006). c-Abl phosphorylates Hdm2 at tyrosine 276 in response to DNA damage and regulates interaction with ARF. Oncogene 25 (50), 6666–6671. 10.1038/sj.onc.1209671 16702947

[B48] Donehower L. A. Harvey M. Slagle B. L. McArthur M. J. Montgomery C. J. Butel J. S. (1992). Mice deficient for p53 are developmentally normal but susceptible to spontaneous tumours. Nature 356 (6366), 215–221. 10.1038/356215a0 1552940

[B49] Eischen C. M. Lozano G. (2014). The Mdm network and its regulation of p53 activities: a rheostat of cancer risk. Hum. Mutat. 35 (6), 728–737. 10.1002/humu.22524 24488925 PMC4725600

[B50] Eischen C. M. Weber J. D. Roussel M. F. Sherr C. J. Cleveland J. L. (1999). Disruption of the ARF-Mdm2-p53 tumor suppressor pathway in Myc-induced lymphomagenesis. Genes Dev. 13 (20), 2658–2669. 10.1101/gad.13.20.2658 10541552 PMC317106

[B51] Eischen C. M. (2017). Role of Mdm2 and Mdmx in DNA repair. J. Mol. Cell Biol. 9 (1), 69–73. 10.1093/jmcb/mjw052 27932484 PMC5439402

[B52] Erba H. P. Becker P. S. Shami P. J. Grunwald M. R. Flesher D. L. Zhu M. (2019). Phase 1b study of the MDM2 inhibitor AMG 232 with or without trametinib in relapsed/refractory acute myeloid leukemia. Blood Adv. 3 (13), 1939–1949. 10.1182/bloodadvances.2019030916 31253596 PMC6616264

[B53] Espadinha M. Barcherini V. Lopes E. A. Santos M. (2018). An Update on MDMX and Dual MDM2/X Inhibitors. Curr. Top. Med. Chem. 18 (8), 647–660. 10.2174/1568026618666180604080119 29866007

[B54] Evans S. C. Viswanathan M. Grier J. D. Narayana M. El-Naggar A. K. Lozano G. (2001). An alternatively spliced HDM2 product increases p53 activity by inhibiting HDM2. Oncogene 20 (30), 4041–4049. 10.1038/sj.onc.1204533 11494132

[B55] Evdokiou A. Atkins G. J. Bouralexis S. Hay S. Raggatt L. J. Cowled P. A. (2001). Expression of alternatively-spliced MDM2 transcripts in giant cell tumours of bone. Int. J. Oncol. 19 (3), 625–632. 10.3892/ijo.19.3.625 11494046

[B56] Fahraeus R. Olivares-Illana V. (2014). MDM2’s social network. Oncogene 33 (35), 4365–4376. 10.1038/onc.2013.410 24096477

[B57] Fang S. Jensen J. P. Ludwig R. L. Vousden K. H. Weissman A. M. (2000). Mdm2 is a RING finger-dependent ubiquitin protein ligase for itself and p53. J. Biol. Chem. 275 (12), 8945–8951. 10.1074/jbc.275.12.8945 10722742

[B58] Fang D. D. Tang Q. Kong Y. Wang Q. Gu J. Fang X. (2019). MDM2 inhibitor APG-115 synergizes with PD-1 blockade through enhancing antitumor immunity in the tumor microenvironment. J. Immunother. Cancer 7 (1), 327. 10.1186/s40425-019-0750-6 31779710 PMC6883539

[B59] Freedman D. A. Wu L. Levine A. J. (1999). Functions of the MDM2 oncoprotein. Cell Mol. Life Sci. 55 (1), 96–107. 10.1007/s000180050273 10065155 PMC11146946

[B60] Frum R. A. Singh S. Vaughan C. Mukhopadhyay N. D. Grossman S. R. Windle B. (2014). The human oncoprotein MDM2 induces replication stress eliciting early intra-S-phase checkpoint response and inhibition of DNA replication origin firing. Nucleic Acids Res. 42 (2), 926–940. 10.1093/nar/gkt944 24163099 PMC3902934

[B61] Ganem N. J. Storchova Z. Pellman D. (2007). Tetraploidy, aneuploidy and cancer. Curr. Opin. Genet. Dev. 17 (2), 157–162. 10.1016/j.gde.2007.02.011 17324569

[B62] Geyer R. K. Yu Z. K. Maki C. G. (2000). The MDM2 RING-finger domain is required to promote p53 nuclear export. Nat. Cell Biol. 2 (9), 569–573. 10.1038/35023507 10980696

[B63] Gill G. (2003). Post-translational modification by the small ubiquitin-related modifier SUMO has big effects on transcription factor activity. Curr. Opin. Genet. Dev. 13 (2), 108–113. 10.1016/s0959-437x(03)00021-2 12672486

[B64] Giustiniano M. Daniele S. Pelliccia S. La Pietra V. Pietrobono D. Brancaccio D. (2017). Computer-Aided Identification and Lead Optimization of Dual Murine Double Minute 2 and 4 Binders: Structure-Activity Relationship Studies and Pharmacological Activity. J. Med. Chem. 60 (19), 8115–8130. 10.1021/acs.jmedchem.7b00912 28921985

[B65] Goldberg Z. Vogt S. R. Berger M. Zwang Y. Perets R. Van Etten R. A. (2002). Tyrosine phosphorylation of Mdm2 by c-Abl: implications for p53 regulation. EMBO J. 21 (14), 3715–3727. 10.1093/emboj/cdf384 12110584 PMC125401

[B66] Gotz C. Kartarius S. Scholtes P. Nastainczyk W. Montenarh M. (1999). Identification of a CK2 phosphorylation site in mdm2. Eur. J. Biochem. 266 (2), 493–501. 10.1046/j.1432-1327.1999.00882.x 10561590

[B67] Gryshchenko I. Hofbauer S. Stoecher M. Daniel P. T. Steurer M. Gaiger A. (2008). MDM2 SNP309 is associated with poor outcome in B-cell chronic lymphocytic leukemia. J. Clin. Oncol. 26 (14), 2252–2257. 10.1200/JCO.2007.11.5212 18467716

[B68] Gu L. Zhang H. Liu T. Zhou S. Du Y. Xiong J. (2016). Discovery of Dual Inhibitors of MDM2 and XIAP for Cancer Treatment. Cancer Cell 30 (4), 623–636. 10.1016/j.ccell.2016.08.015 27666947 PMC5079537

[B69] Harper J. W. Elledge S. J. (2007). The DNA damage response: Ten years after. Mol. Cell 28 (5), 739–745. 10.1016/j.molcel.2007.11.015 18082599

[B70] Haupt Y. Maya R. Kazaz A. Oren M. (1997). Mdm2 promotes the rapid degradation of p53. Nature 387 (6630), 296–299. 10.1038/387296a0 9153395

[B71] He S. Dong G. Wu S. Fang K. Miao Z. Wang W. (2018). Small Molecules Simultaneously Inhibiting p53-Murine Double Minute 2 (MDM2) Interaction and Histone Deacetylases (HDACs): Discovery of Novel Multitargeting Antitumor Agents. J. Med. Chem. 61 (16), 7245–7260. 10.1021/acs.jmedchem.8b00664 30045621

[B72] Hirata H. Hinoda Y. Kikuno N. Kawamoto K. Suehiro Y. Tanaka Y. (2007). MDM2 SNP309 polymorphism as risk factor for susceptibility and poor prognosis in renal cell carcinoma. Clin. Cancer Res. 13 (14), 4123–4129. 10.1158/1078-0432.CCR-07-0609 17634539

[B73] Hjerrild M. Milne D. Dumaz N. Hay T. Issinger O. G. Meek D. (2001). Phosphorylation of murine double minute clone 2 (MDM2) protein at serine-267 by protein kinase CK2 in vitro and in cultured cells. Biochem. J. 355 (Pt 2), 347–356. 10.1042/0264-6021:3550347 11284721 PMC1221745

[B74] Hoeijmakers J. H. (2009). DNA damage, aging, and cancer. N Engl. J. Med. 361 (15), 1475–1485. 10.1056/NEJMra0804615 19812404

[B75] Honda R. Yasuda H. (2000). Activity of MDM2, a ubiquitin ligase, toward p53 or itself is dependent on the RING finger domain of the ligase. Oncogene 19 (11), 1473–1476. 10.1038/sj.onc.1203464 10723139

[B76] Hong Y. Miao X. Zhang X. Ding F. Luo A. Guo Y. (2005). The role of P53 and MDM2 polymorphisms in the risk of esophageal squamous cell carcinoma. Cancer Res. 65 (20), 9582–9587. 10.1158/0008-5472.CAN-05-1460 16230424

[B77] Huun J. Gansmo L. B. Mannsaker B. Iversen G. T. Ovrebo J. I. Lonning P. E. (2017). Impact of the MDM2 splice-variants MDM2-A, MDM2-B and MDM2-C on cytotoxic stress response in breast cancer cells. BMC Cell Biol. 18 (1), 17. 10.1186/s12860-017-0134-z 28415963 PMC5393014

[B78] Itahana K. Mao H. Jin A. Itahana Y. Clegg H. V. Lindstrom M. S. (2007). Targeted inactivation of Mdm2 RING finger E3 ubiquitin ligase activity in the mouse reveals mechanistic insights into p53 regulation. Cancer Cell 12 (4), 355–366. 10.1016/j.ccr.2007.09.007 17936560

[B79] Jacks T. Remington L. Williams B. O. Schmitt E. M. Halachmi S. Bronson R. T. (1994). Tumor spectrum analysis in p53-mutant mice. Curr. Biol. 4 (1), 1–7. 10.1016/s0960-9822(00)00002-6 7922305

[B80] Jackson S. P. Bartek J. (2009). The DNA-damage response in human biology and disease. Nature 461 (7267), 1071–1078. 10.1038/nature08467 19847258 PMC2906700

[B81] Jasin M. (2000). Chromosome breaks and genomic instability. Cancer Invest. 18 (1), 78–86. 10.3109/07357900009023065 10701370

[B82] Jeay S. Ferretti S. Holzer P. Fuchs J. Chapeau E. A. Wartmann M. (2018). Dose and Schedule Determine Distinct Molecular Mechanisms Underlying the Efficacy of the p53-MDM2 Inhibitor HDM201. Cancer Res. 78 (21), 6257–6267. 10.1158/0008-5472.CAN-18-0338 30135191

[B83] Jeyaraj S. O’Brien D. M. Chandler D. S. (2009). MDM2 and MDM4 splicing: an integral part of the cancer spliceome. Front. Biosci. (Landmark Ed) 14, 2647–2656. 10.2741/3402 19273224

[B84] Jones S. N. Roe A. E. Donehower L. A. Bradley A. (1995). Rescue of embryonic lethality in Mdm2-deficient mice by absence of p53. Nature 378 (6553), 206–208. 10.1038/378206a0 7477327

[B85] Jones S. N. Hancock A. R. Vogel H. Donehower L. A. Bradley A. (1998). Overexpression of Mdm2 in mice reveals a p53-independent role for Mdm2 in tumorigenesis. Proc. Natl. Acad. Sci. U. S. A 95 (26), 15608–15612. 10.1073/pnas.95.26.15608 9861017 PMC28091

[B86] Kang M. H. Reynolds C. P. Kolb E. A. Gorlick R. Carol H. Lock R. (2016). Initial Testing (Stage 1) of MK-8242-A Novel MDM2 Inhibitor-by the Pediatric Preclinical Testing Program. Pediatr. Blood Cancer 63 (10), 1744–1752. 10.1002/pbc.26064 27238606 PMC5657425

[B87] Kimura S. H. Nojima H. (2002). Cyclin G1 associates with MDM2 and regulates accumulation and degradation of p53 protein. Genes Cells 7 (8), 869–880. 10.1046/j.1365-2443.2002.00564.x 12167164

[B88] Kouzarides T. (2007). Chromatin modifications and their function. Cell 128 (4), 693–705. 10.1016/j.cell.2007.02.005 17320507

[B89] Kruse J. P. Gu W. (2009). Modes of p53 regulation. Cell 137 (4), 609–622. 10.1016/j.cell.2009.04.050 19450511 PMC3737742

[B90] Kubbutat M. H. Jones S. N. Vousden K. H. (1997). Regulation of p53 stability by Mdm2. Nature 387 (6630), 299–303. 10.1038/387299a0 9153396

[B91] Ladanyi M. Cha C. Lewis R. Jhanwar S. C. Huvos A. G. Healey J. H. (1993). MDM2 gene amplification in metastatic osteosarcoma. Cancer Res. 53 (1), 16–18. 8416741

[B92] Lai Z. Ferry K. V. Diamond M. A. Wee K. E. Kim Y. B. Ma J. (2001). Human mdm2 mediates multiple mono-ubiquitination of p53 by a mechanism requiring enzyme isomerization. J. Biol. Chem. 276 (33), 31357–31367. 10.1074/jbc.M011517200 11397792

[B93] Lawlor M. A. Alessi D. R. (2001). PKB/Akt: a key mediator of cell proliferation, survival and insulin responses? J. Cell Sci. 114 (Pt 16), 2903–2910. 11686294 10.1242/jcs.114.16.2903

[B94] Lee S. Elenbaas B. Levine A. Griffith J. (1995). p53 and its 14 kDa C-terminal domain recognize primary DNA damage in the form of insertion/deletion mismatches. Cell 81 (7), 1013–1020. 10.1016/s0092-8674(05)80006-6 7600570

[B95] Li M. Brooks C. L. Wu-Baer F. Chen D. Baer R. Gu W. (2003). Mono- versus polyubiquitination: differential control of p53 fate by Mdm2. Science 302 (5652), 1972–1975. 10.1126/science.1091362 14671306

[B96] Lin J. Chen J. Elenbaas B. Levine A. J. (1994). Several hydrophobic amino acids in the p53 amino-terminal domain are required for transcriptional activation, binding to mdm-2 and the adenovirus 5 E1B 55-kD protein. Genes Dev. 8 (10), 1235–1246. 10.1101/gad.8.10.1235 7926727

[B97] Linares L. K. Kiernan R. Triboulet R. Chable-Bessia C. Latreille D. Cuvier O. (2007). Intrinsic ubiquitination activity of PCAF controls the stability of the oncoprotein Hdm2. Nat. Cell Biol. 9 (3), 331–338. 10.1038/ncb1545 17293853

[B98] Liu Y. Kulesz-Martin M. (2001). p53 protein at the hub of cellular DNA damage response pathways through sequence-specific and non-sequence-specific DNA binding. Carcinogenesis 22 (6), 851–860. 10.1093/carcin/22.6.851 11375889

[B99] Liu T. Zhang H. Xiong J. Yi S. Gu L. Zhou M. (2015). Inhibition of MDM2 homodimerization by XIAP IRES stabilizes MDM2, influencing cancer cell survival. Mol. Cancer 14, 65. 10.1186/s12943-015-0334-0 25888903 PMC4379586

[B100] Liu Y. Wang X. Wang G. Yang Y. Yuan Y. Ouyang L. (2019). The past, present and future of potential small-molecule drugs targeting p53-MDM2/MDMX for cancer therapy. Eur. J. Med. Chem. 176, 92–104. 10.1016/j.ejmech.2019.05.018 31100649

[B101] Lohrum M. A. Woods D. B. Ludwig R. L. Balint E. Vousden K. H. (2001). C-terminal ubiquitination of p53 contributes to nuclear export. Mol. Cell Biol. 21 (24), 8521–8532. 10.1128/MCB.21.24.8521-8532.2001 11713287 PMC100015

[B102] Lu H. Levine A. J. (1995). Human TAFII31 protein is a transcriptional coactivator of the p53 protein. Proc. Natl. Acad. Sci. U. S. A 92 (11), 5154–5158. 10.1073/pnas.92.11.5154 7761466 PMC41867

[B103] Lu X. Ma O. Nguyen T. A. Jones S. N. Oren M. Donehower L. A. (2007). The Wip1 Phosphatase acts as a gatekeeper in the p53-Mdm2 autoregulatory loop. Cancer Cell 12 (4), 342–354. 10.1016/j.ccr.2007.08.033 17936559

[B104] Lukas J. Gao D. Q. Keshmeshian M. Wen W. H. Tsao-Wei D. Rosenberg S. (2001). Alternative and aberrant messenger RNA splicing of the mdm2 oncogene in invasive breast cancer. Cancer Res. 61 (7), 3212–3219. 11306511

[B105] Lundgren K. Montes D. O. L. R. McNeill Y. B. Emerick E. P. Spencer B. Barfield C. R. (1997). Targeted expression of MDM2 uncouples S phase from mitosis and inhibits mammary gland development independent of p53. Genes Dev. 11 (6), 714–725. 10.1101/gad.11.6.714 9087426

[B106] Lushnikova T. Bouska A. Odvody J. Dupont W. D. Eischen C. M. (2011). Aging mice have increased chromosome instability that is exacerbated by elevated Mdm2 expression. Oncogene 30 (46), 4622–4631. 10.1038/onc.2011.172 21602883 PMC3161162

[B107] Maglott A. Bartik P. Cosgun S. Klotz P. Ronde P. Fuhrmann G. (2006). The small alpha5beta1 integrin antagonist, SJ749, reduces proliferation and clonogenicity of human astrocytoma cells. Cancer Res. 66 (12), 6002–6007. 10.1158/0008-5472.CAN-05-4105 16778170

[B108] Maltzman W. Czyzyk L. (1984). UV irradiation stimulates levels of p53 cellular tumor antigen in nontransformed mouse cells. Mol. Cell Biol. 4 (9), 1689–1694. 10.1128/mcb.4.9.1689 6092932 PMC368974

[B109] Mann B. S. Johnson J. R. Cohen M. H. Justice R. Pazdur R. (2007). FDA approval summary: vorinostat for treatment of advanced primary cutaneous T-cell lymphoma. Oncologist 12 (10), 1247–1252. 10.1634/theoncologist.12-10-1247 17962618

[B110] Mas-Moruno C. Rechenmacher F. Kessler H. (2010). Cilengitide: the first anti-angiogenic small molecule drug candidate design, synthesis and clinical evaluation. Anticancer Agents Med. Chem. 10 (10), 753–768. 10.2174/187152010794728639 21269250 PMC3267166

[B111] Matsumoto R. Tada M. Nozaki M. Zhang C. L. Sawamura Y. Abe H. (1998). Short alternative splice transcripts of the mdm2 oncogene correlate to malignancy in human astrocytic neoplasms. Cancer Res. 58 (4), 609–613. 9485008

[B112] Maya R. Balass M. Kim S. T. Shkedy D. Leal J. F. Shifman O. (2001). ATM-dependent phosphorylation of Mdm2 on serine 395: role in p53 activation by DNA damage. Genes Dev. 15 (9), 1067–1077. 10.1101/gad.886901 11331603 PMC312683

[B113] Mayo L. D. Donner D. B. (2001). A phosphatidylinositol 3-kinase/Akt pathway promotes translocation of Mdm2 from the cytoplasm to the nucleus. Proc. Natl. Acad. Sci. U. S. A 98 (20), 11598–11603. 10.1073/pnas.181181198 11504915 PMC58775

[B114] Mayo L. D. Donner D. B. (2002). The PTEN, Mdm2, p53 tumor suppressor-oncoprotein network. Trends Biochem. Sci. 27 (9), 462–467. 10.1016/s0968-0004(02)02166-7 12217521

[B115] Mayo L. D. Turchi J. J. Berberich S. J. (1997). Mdm-2 phosphorylation by DNA-dependent protein kinase prevents interaction with p53. Cancer Res. 57 (22), 5013–5016. 9371494

[B116] Mayo L. D. Dixon J. E. Durden D. L. Tonks N. K. Donner D. B. (2002). PTEN protects p53 from Mdm2 and sensitizes cancer cells to chemotherapy. J. Biol. Chem. 277 (7), 5484–5489. 10.1074/jbc.M108302200 11729185

[B117] McCormack E. Haaland I. Venas G. Forthun R. B. Huseby S. Gausdal G. (2012). Synergistic induction of p53 mediated apoptosis by valproic acid and nutlin-3 in acute myeloid leukemia. Leukemia 26 (5), 910–917. 10.1038/leu.2011.315 22064349

[B118] McCoy M. A. Gesell J. J. Senior M. M. Wyss D. F. (2003). Flexible lid to the p53-binding domain of human Mdm2: implications for p53 regulation. Proc. Natl. Acad. Sci. U. S. A 100 (4), 1645–1648. 10.1073/pnas.0334477100 12552135 PMC149886

[B119] Meek D. W. Hupp T. R. (2010). The regulation of MDM2 by multisite phosphorylation–opportunities for molecular-based intervention to target tumours? Semin. Cancer Biol. 20 (1), 19–28. 10.1016/j.semcancer.2009.10.005 19897041

[B120] Meek D. W. Knippschild U. (2003). Posttranslational modification of MDM2. Mol. Cancer Res. 1 (14), 1017–1026. 14707285

[B121] Mendrysa S. M. McElwee M. K. Michalowski J. O’Leary K. A. Young K. M. Perry M. E. (2003). mdm2 Is critical for inhibition of p53 during lymphopoiesis and the response to ionizing irradiation. Mol. Cell Biol. 23 (2), 462–472. 10.1128/mcb.23.2.462-473.2003 12509446 PMC151546

[B122] Merlino F. Daniele S. La Pietra V. Di Maro S. Di Leva F. S. Brancaccio D. (2018). Simultaneous Targeting of RGD-Integrins and Dual Murine Double Minute Proteins in Glioblastoma Multiforme. J. Med. Chem. 61 (11), 4791–4809. 10.1021/acs.jmedchem.8b00004 29775303

[B123] Minsky N. Oren M. (2004). The RING domain of Mdm2 mediates histone ubiquitylation and transcriptional repression. Mol. Cell 16 (4), 631–639. 10.1016/j.molcel.2004.10.016 15546622

[B124] Mirzayans R. Enns L. Dietrich K. Barley R. D. Paterson M. C. (1996). Faulty DNA polymerase delta/epsilon-mediated excision repair in response to gamma radiation or ultraviolet light in p53-deficient fibroblast strains from affected members of a cancer-prone family with Li-Fraumeni syndrome. Carcinogenesis 17 (4), 691–698. 10.1093/carcin/17.4.691 8625479

[B125] Mizutani Y. Nakanishi H. Li Y. N. Matsubara H. Yamamoto K. Sato N. (2007). Overexpression of XIAP expression in renal cell carcinoma predicts a worse prognosis. Int. J. Oncol. 30 (4), 919–925. 10.3892/ijo.30.4.919 17332931

[B126] Mladenov E. Magin S. Soni A. Iliakis G. (2016). DNA double-strand-break repair in higher eukaryotes and its role in genomic instability and cancer: Cell cycle and proliferation-dependent regulation. Semin. Cancer Biol. 37-38, 51–64. 10.1016/j.semcancer.2016.03.003 27016036

[B127] Momand J. Zambetti G. P. Olson D. C. George D. Levine A. J. (1992). The mdm-2 oncogene product forms a complex with the p53 protein and inhibits p53-mediated transactivation. Cell 69 (7), 1237–1245. 10.1016/0092-8674(92)90644-r 1535557

[B128] Montes D. O. L. R. Wagner D. S. Lozano G. (1995). Rescue of early embryonic lethality in mdm2-deficient mice by deletion of p53. Nature 378 (6553), 203–206. 10.1038/378203a0 7477326

[B129] Montesinos P. Beckermann B. M. Catalani O. Esteve J. Gamel K. Konopleva M. Y. (2020). MIRROS: a randomized, placebo-controlled, Phase III trial of cytarabine +/- idasanutlin in relapsed or refractory acute myeloid leukemia. Future Oncol. 16 (13), 807–815. 10.2217/fon-2020-0044 32167393

[B130] Morgan W. F. Corcoran J. Hartmann A. Kaplan M. I. Limoli C. L. Ponnaiya B. (1998). DNA double-strand breaks, chromosomal rearrangements, and genomic instability. Mutat. Res. 404 (1-2), 125–128. 10.1016/s0027-5107(98)00104-3 9729329

[B131] Ogawara Y. Kishishita S. Obata T. Isazawa Y. Suzuki T. Tanaka K. (2002). Akt enhances Mdm2-mediated ubiquitination and degradation of p53. J. Biol. Chem. 277 (24), 21843–21850. 10.1074/jbc.M109745200 11923280

[B132] Okamoto K. Li H. Jensen M. R. Zhang T. Taya Y. Thorgeirsson S. S. (2002). Cyclin G recruits PP2A to dephosphorylate Mdm2. Mol. Cell 9 (4), 761–771. 10.1016/s1097-2765(02)00504-x 11983168

[B133] Okoro D. R. Arva N. Gao C. Polotskaia A. Puente C. Rosso M. (2013). Endogenous human MDM2-C is highly expressed in human cancers and functions as a p53-independent growth activator. PloS One 8 (10), e77643. 10.1371/journal.pone.0077643 24147044 PMC3795673

[B134] Oliner J. D. Kinzler K. W. Meltzer P. S. George D. L. Vogelstein B. (1992). Amplification of a gene encoding a p53-associated protein in human sarcomas. Nature 358 (6381), 80–83. 10.1038/358080a0 1614537

[B135] Oliner J. D. Pietenpol J. A. Thiagalingam S. Gyuris J. Kinzler K. W. Vogelstein B. (1993). Oncoprotein MDM2 conceals the activation domain of tumour suppressor p53. Nature 362 (6423), 857–860. 10.1038/362857a0 8479525

[B136] Pahl H. L. (1999). Activators and target genes of Rel/NF-kappaB transcription factors. Oncogene 18 (49), 6853–6866. 10.1038/sj.onc.1203239 10602461

[B137] Patnaik A. Tolcher A. Beeram M. Nemunaitis J. Weiss G. J. Bhalla K. (2015). Clinical pharmacology characterization of RG7112, an MDM2 antagonist, in patients with advanced solid tumors. Cancer Chemother. Pharmacol. 76 (3), 587–595. 10.1007/s00280-015-2830-8 26210682

[B138] Paull T. T. Gellert M. (1999). Nbs1 potentiates ATP-driven DNA unwinding and endonuclease cleavage by the Mre11/Rad50 complex. Genes Dev. 13 (10), 1276–1288. 10.1101/gad.13.10.1276 10346816 PMC316715

[B139] Pichiorri F. Suh S. S. Rocci A. De Luca L. Taccioli C. Santhanam R. (2010). Downregulation of p53-inducible microRNAs 192, 194, and 215 impairs the p53/MDM2 autoregulatory loop in multiple myeloma development. Cancer Cell 18 (4), 367–381. 10.1016/j.ccr.2010.09.005 20951946 PMC3561766

[B140] Primo L. Teixeira L. K. (2019). DNA replication stress: oncogenes in the spotlight. Genet. Mol. Biol. 43 (1 suppl 1), e20190138. 10.1590/1678-4685GMB-2019-0138 31930281 PMC7197996

[B141] Purdie C. A. Harrison D. J. Peter A. Dobbie L. White S. Howie S. E. (1994). Tumour incidence, spectrum and ploidy in mice with a large deletion in the p53 gene. Oncogene 9 (2), 603–609. 8290271

[B142] Ranaweera R. S. Yang X. (2013). Auto-ubiquitination of Mdm2 enhances its substrate ubiquitin ligase activity. J. Biol. Chem. 288 (26), 18939–18946. 10.1074/jbc.M113.454470 23671280 PMC3696669

[B143] Ravandi F. Gojo I. Patnaik M. M. Minden M. D. Kantarjian H. Johnson-Levonas A. O. (2016). A phase I trial of the human double minute 2 inhibitor (MK-8242) in patients with refractory/recurrent acute myelogenous leukemia (AML). Leuk. Res. 48, 92–100. 10.1016/j.leukres.2016.07.004 27544076 PMC5408350

[B144] Reis B. Jukofsky L. Chen G. Martinelli G. Zhong H. So W. V. (2016). Acute myeloid leukemia patients’ clinical response to idasanutlin (RG7388) is associated with pre-treatment MDM2 protein expression in leukemic blasts. Haematologica 101 (5), e185–e188. 10.3324/haematol.2015.139717 26869629 PMC5004378

[B145] Rosso M. Okoro D. E. Bargonetti J. (2014). Splice variants of MDM2 in oncogenesis. Subcell. Biochem. 85, 247–261. 10.1007/978-94-017-9211-0_14 25201199

[B146] Sanchez-Aguilera A. Garcia J. F. Sanchez-Beato M. Piris M. A. (2006). Hodgkin’s lymphoma cells express alternatively spliced forms of HDM2 with multiple effects on cell cycle control. Oncogene 25 (18), 2565–2574. 10.1038/sj.onc.1209282 16331255

[B147] Seipel K. Marques M. Sidler C. Mueller B. U. Pabst T. (2018). MDM2- and FLT3-inhibitors in the treatment of FLT3-ITD acute myeloid leukemia, specificity and efficacy of NVP-HDM201 and midostaurin. Haematologica 103 (11), 1862–1872. 10.3324/haematol.2018.191650 29976747 PMC6278968

[B148] Senturk E. Manfredi J. J. (2012). Mdm2 and tumorigenesis: evolving theories and unsolved mysteries. Genes Cancer 3 (3-4), 192–198. 10.1177/1947601912457368 23150752 PMC3494366

[B149] Shafman T. Khanna K. K. Kedar P. Spring K. Kozlov S. Yen T. (1997). Interaction between ATM protein and c-Abl in response to DNA damage. Nature 387 (6632), 520–523. 10.1038/387520a0 9168117

[B150] Shieh S. Y. Ikeda M. Taya Y. Prives C. (1997). DNA damage-induced phosphorylation of p53 alleviates inhibition by MDM2. Cell 91 (3), 325–334. 10.1016/s0092-8674(00)80416-x 9363941

[B151] Shirai Y. Shiba H. Iwase R. Haruki K. Fujiwara Y. Furukawa K. (2016). Dual inhibition of nuclear factor kappa-B and Mdm2 enhance the antitumor effect of radiation therapy for pancreatic cancer. Cancer Lett. 370 (2), 177–184. 10.1016/j.canlet.2015.10.034 26546875

[B152] Sigalas I. Calvert A. H. Anderson J. J. Neal D. E. Lunec J. (1996). Alternatively spliced mdm2 transcripts with loss of p53 binding domain sequences: transforming ability and frequent detection in human cancer. Nat. Med. 2 (8), 912–917. 10.1038/nm0896-912 8705862

[B153] Sionov R. V. Coen S. Goldberg Z. Berger M. Bercovich B. Ben-Neriah Y. (2001). c-Abl regulates p53 levels under normal and stress conditions by preventing its nuclear export and ubiquitination. Mol. Cell Biol. 21 (17), 5869–5878. 10.1128/mcb.21.17.5869-5878.2001 11486026 PMC87306

[B154] Skalniak L. Kocik J. Polak J. Skalniak A. Rak M. Wolnicka-Glubisz A. (2018). Prolonged Idasanutlin (RG7388) Treatment Leads to the Generation of p53-Mutated Cells. Cancers (Basel) 10 (11), 396. 10.3390/cancers10110396 PMC6266412 30352966

[B155] Smith M. L. Chen I. T. Zhan Q. O’Connor P. M. Fornace A. J. (1995). Involvement of the p53 tumor suppressor in repair of u.v.-type DNA damage. Oncogene 10 (6), 1053–1059. 7700629

[B156] Soutoglou E. Misteli T. (2010). Activation of the cellular DNA damage response in the absence of DNA lesions (vol 320, pg 1507, 2008). Science 327 (5968), 959. 10.1126/science.1159051 PMC2575099 18483401

[B157] Stommel J. M. Wahl G. M. (2004). Accelerated MDM2 auto-degradation induced by DNA-damage kinases is required for p53 activation. EMBO J. 23 (7), 1547–1556. 10.1038/sj.emboj.7600145 15029243 PMC391059

[B158] Stommel J. M. Wahl G. M. (2005). A new twist in the feedback loop: stress-activated MDM2 destabilization is required for p53 activation. Cell Cycle 4 (3), 411–417. 10.4161/cc.4.3.1522 15684615

[B159] Stracker T. H. Theunissen J. W. Morales M. Petrini J. H. (2004). The Mre11 complex and the metabolism of chromosome breaks: the importance of communicating and holding things together. DNA Repair (Amst) 3 (8-9), 845–854. 10.1016/j.dnarep.2004.03.014 15279769

[B160] Sui G. Affar E. B. Shi Y. Brignone C. Wall N. R. Yin P. (2004). Yin Yang 1 is a negative regulator of p53. Cell 117 (7), 859–872. 10.1016/j.cell.2004.06.004 15210108

[B161] Sullivan K. D. Galbraith M. D. Andrysik Z. Espinosa J. M. (2018). Mechanisms of transcriptional regulation by p53. Cell Death Differ. 25 (1), 133–143. 10.1038/cdd.2017.174 29125602 PMC5729533

[B162] Sun D. Li Z. Rew Y. Gribble M. Bartberger M. D. Beck H. P. (2014). Discovery of AMG 232, a potent, selective, and orally bioavailable MDM2-p53 inhibitor in clinical development. J. Med. Chem. 57 (4), 1454–1472. 10.1021/jm401753e 24456472

[B163] Tait S. W. Green D. R. (2010). Mitochondria and cell death: outer membrane permeabilization and beyond. Nat. Rev. Mol. Cell Biol. 11 (9), 621–632. 10.1038/nrm2952 20683470

[B164] Takahashi Y. Kitadai Y. Bucana C. D. Cleary K. R. Ellis L. M. (1995). Expression of vascular endothelial growth factor and its receptor, KDR, correlates with vascularity, metastasis, and proliferation of human colon cancer. Cancer Res. 55 (18), 3964–3968. 7664263

[B165] Tamm I. Richter S. Oltersdorf D. Creutzig U. Harbott J. Scholz F. (2004). High expression levels of x-linked inhibitor of apoptosis protein and survivin correlate with poor overall survival in childhood de novo acute myeloid leukemia. Clin. Cancer Res. 10 (11), 3737–3744. 10.1158/1078-0432.CCR-03-0642 15173080

[B166] Tan J. Cang S. Ma Y. Petrillo R. L. Liu D. (2010). Novel histone deacetylase inhibitors in clinical trials as anti-cancer agents. J. Hematol. Oncol. 3, 5. 10.1186/1756-8722-3-5 20132536 PMC2827364

[B167] Tang Y. Zhao W. Chen Y. Zhao Y. Gu W. (2008). Acetylation is indispensable for p53 activation. Cell 133 (4), 612–626. 10.1016/j.cell.2008.03.025 18485870 PMC2914560

[B168] Teveroni E. Luca R. Pellegrino M. Ciolli G. Pontecorvi A. Moretti F. (2016). Peptides and peptidomimetics in the p53/MDM2/MDM4 circuitry - a patent review. Expert Opin. Ther. Pat. 26 (12), 1417–1429. 10.1080/13543776.2017.1233179 27603098

[B169] Thut C. J. Chen J. L. Klemm R. Tjian R. (1995). p53 transcriptional activation mediated by coactivators TAFII40 and TAFII60. Science 267 (5194), 100–104. 10.1126/science.7809597 7809597

[B170] Thut C. J. Goodrich J. A. Tjian R. (1997). Repression of p53-mediated transcription by MDM2: a dual mechanism. Genes Dev. 11 (15), 1974–1986. 10.1101/gad.11.15.1974 9271120 PMC316412

[B171] Tolcher A. W. Fang D. D. Li Y. Tang Y. Ji J. Wang H. (2019). 2OA phase Ib/II study of APG-115 in combination with pembrolizumab in patients with unresectable or metastatic melanomas or advanced solid tumors. Ann. Oncol. 30, 1. 10.1093/annonc/mdz027

[B172] Valentin-Vega Y. A. Barboza J. A. Chau G. P. El-Naggar A. K. Lozano G. (2007). High levels of the p53 inhibitor MDM4 in head and neck squamous carcinomas. Hum. Pathol. 38 (10), 1553–1562. 10.1016/j.humpath.2007.03.005 17651783 PMC2699677

[B173] Vanhaesebroeck B. Alessi D. R. (2000). The PI3K-PDK1 connection: more than just a road to PKB. Biochem. J. 346 Pt 3, 561–576. 10.1042/0264-6021:3460561 10698680 PMC1220886

[B174] Vassilev L. T. Vu B. T. Graves B. Carvajal D. Podlaski F. Filipovic Z. (2004). In vivo activation of the p53 pathway by small-molecule antagonists of MDM2. Science 303 (5659), 844–848. 10.1126/science.1092472 14704432

[B175] Vazquez A. Bond E. E. Levine A. J. Bond G. L. (2008). The genetics of the p53 pathway, apoptosis and cancer therapy. Nat. Rev. Drug Discovery 7 (12), 979–987. 10.1038/nrd2656 19043449

[B176] Verreault M. Schmitt C. Goldwirt L. Pelton K. Haidar S. Levasseur C. (2016). Preclinical Efficacy of the MDM2 Inhibitor RG7112 in MDM2-Amplified and TP53 Wild-type Glioblastomas. Clin. Cancer Res. 22 (5), 1185–1196. 10.1158/1078-0432.CCR-15-1015 26482041 PMC4842012

[B177] Volk E. L. Fan L. Schuster K. Rehg J. E. Harris L. C. (2009). The MDM2-a splice variant of MDM2 alters transformation in vitro and the tumor spectrum in both Arf- and p53-null models of tumorigenesis. Mol. Cancer Res. 7 (6), 863–869. 10.1158/1541-7786.MCR-08-0418 19491200 PMC3526663

[B178] Vu B. Wovkulich P. Pizzolato G. Lovey A. Ding Q. Jiang N. (2013). Discovery of RG7112: A Small-Molecule MDM2 Inhibitor in Clinical Development. ACS Med. Chem. Lett. 4 (5), 466–469. 10.1021/ml4000657 24900694 PMC4027145

[B179] Wade M. Li Y. C. Wahl G. M. (2013). MDM2, MDMX and p53 in oncogenesis and cancer therapy. Nat. Rev. Cancer 13 (2), 83–96. 10.1038/nrc3430 23303139 PMC4161369

[B180] Wagner A. J. Banerji U. Mahipal A. Somaiah N. Hirsch H. Fancourt C. (2017). Phase I Trial of the Human Double Minute 2 Inhibitor MK-8242 in Patients With Advanced Solid Tumors. J. Clin. Oncol. 35 (12), 1304–1311. 10.1200/JCO.2016.70.7117 28240971 PMC5946729

[B181] Wang P. Lushnikova T. Odvody J. Greiner T. C. Jones S. N. Eischen C. M. (2008). Elevated Mdm2 expression induces chromosomal instability and confers a survival and growth advantage to B cells. Oncogene 27 (11), 1590–1598. 10.1038/sj.onc.1210788 17828300

[B182] Wang S. P. Wang W. L. Chang Y. L. Wu C. T. Chao Y. C. Kao S. H. (2009). p53 controls cancer cell invasion by inducing the MDM2-mediated degradation of Slug. Nat. Cell Biol. 11 (6), 694–704. 10.1038/ncb1875 19448627

[B183] Wang S. Sun W. Zhao Y. McEachern D. Meaux I. Barriere C. (2014). SAR405838: an optimized inhibitor of MDM2-p53 interaction that induces complete and durable tumor regression. Cancer Res. 74 (20), 5855–5865. 10.1158/0008-5472.CAN-14-0799 25145672 PMC4247201

[B184] Weber J. D. Taylor L. J. Roussel M. F. Sherr C. J. Bar-Sagi D. (1999). Nucleolar Arf sequesters Mdm2 and activates p53. Nat. Cell Biol. 1 (1), 20–26. 10.1038/8991 10559859

[B185] Wei X. Yu Z. K. Ramalingam A. Grossman S. R. Yu J. H. Bloch D. B. (2003). Physical and functional interactions between PML and MDM2. J. Biol. Chem. 278 (31), 29288–29297. 10.1074/jbc.M212215200 12759344

[B186] Welsch M. E. Snyder S. A. Stockwell B. R. (2010). Privileged scaffolds for library design and drug discovery. Curr. Opin. Chem. Biol. 14 (3), 347–361. 10.1016/j.cbpa.2010.02.018 20303320 PMC2908274

[B187] Wienken M. Moll U. M. Dobbelstein M. (2017). Mdm2 as a chromatin modifier. J. Mol. Cell Biol. 9 (1), 74–80. 10.1093/jmcb/mjw046 27927750 PMC5439376

[B188] Williams A. B. Schumacher B. (2016). p53 in the DNA-Damage-Repair Process. Cold Spring Harb. Perspect. Med. 6 (5). 10.1101/cshperspect.a026070 PMC4852800 27048304

[B189] Williams R. S. Williams J. S. Tainer J. A. (2007). Mre11-Rad50-Nbs1 is a keystone complex connecting DNA repair machinery, double-strand break signaling, and the chromatin template. Biochem. Cell Biol. 85 (4), 509–520. 10.1139/O07-069 17713585

[B190] Xiao Z. X. Chen J. Levine A. J. Modjtahedi N. Xing J. Sellers W. R. (1995). Interaction between the retinoblastoma protein and the oncoprotein MDM2. Nature 375 (6533), 694–698. 10.1038/375694a0 7791904

[B191] Xirodimas D. P. Saville M. K. Bourdon J. C. Hay R. T. Lane D. P. (2004). Mdm2-mediated NEDD8 conjugation of p53 inhibits its transcriptional activity. Cell 118 (1), 83–97. 10.1016/j.cell.2004.06.016 15242646

[B192] Xu W. S. Parmigiani R. B. Marks P. A. (2007). Histone deacetylase inhibitors: molecular mechanisms of action. Oncogene 26 (37), 5541–5552. 10.1038/sj.onc.1210620 17694093

[B193] Yang J. Y. Zong C. S. Xia W. Wei Y. Ali-Seyed M. Li Z. (2006). MDM2 promotes cell motility and invasiveness by regulating E-cadherin degradation. Mol. Cell Biol. 26 (19), 7269–7282. 10.1128/MCB.00172-06 16980628 PMC1592879

[B194] Yang J. Y. Zong C. S. Xia W. Yamaguchi H. Ding Q. Xie X. (2008). ERK promotes tumorigenesis by inhibiting FOXO3a via MDM2-mediated degradation. Nat. Cell Biol. 10 (2), 138–148. 10.1038/ncb1676 18204439 PMC2376808

[B195] Zauberman A. Flusberg D. Haupt Y. Barak Y. Oren M. (1995). A functional p53-responsive intronic promoter is contained within the human mdm2 gene. Nucleic Acids Res. 23 (14), 2584–2592. 10.1093/nar/23.14.2584 7651818 PMC307078

[B196] Zhang L. Hill R. P. (2004). Hypoxia enhances metastatic efficiency by up-regulating Mdm2 in KHT cells and increasing resistance to apoptosis. Cancer Res. 64 (12), 4180–4189. 10.1158/0008-5472.CAN-03-3038 15205329

[B197] Zhang Y. Xiong Y. Yarbrough W. G. (1998). ARF promotes MDM2 degradation and stabilizes p53: ARF-INK4a locus deletion impairs both the Rb and p53 tumor suppression pathways. Cell 92 (6), 725–734. 10.1016/s0092-8674(00)81401-4 9529249

[B198] Zhou B. P. Liao Y. Xia W. Zou Y. Spohn B. Hung M. C. (2001). HER-2/neu induces p53 ubiquitination via Akt-mediated MDM2 phosphorylation. Nat. Cell Biol. 3 (11), 973–982. 10.1038/ncb1101-973 11715018

[B199] Zhuang C. Miao Z. Wu Y. Guo Z. Li J. Yao J. (2014). Double-edged swords as cancer therapeutics: novel, orally active, small molecules simultaneously inhibit p53-MDM2 interaction and the NF-kappaB pathway. J. Med. Chem. 57 (3), 567–577. 10.1021/jm401800k 24428757

[B200] Zietz C. Rossle M. Haas C. Sendelhofert A. Hirschmann A. Sturzl M. (1998). MDM-2 oncoprotein overexpression, p53 gene mutation, and VEGF up-regulation in angiosarcomas. Am. J. Pathol. 153 (5), 1425–1433. 10.1016/S0002-9440(10)65729-X 9811333 PMC1876718

[B201] Zindy F. Eischen C. M. Randle D. H. Kamijo T. Cleveland J. L. Sherr C. J. (1998). Myc signaling via the ARF tumor suppressor regulates p53-dependent apoptosis and immortalization. Genes Dev. 12 (15), 2424–2433. 10.1101/gad.12.15.2424 9694806 PMC317045

